# Effectiveness of abdominal bracing core exercises as rehabilitation therapy for reducing abdominal symptoms in patients with autosomal dominant polycystic kidney disease and significant polycystic liver disease

**DOI:** 10.1080/0886022X.2025.2457519

**Published:** 2025-03-11

**Authors:** Jaeyeong Yoo, Jin Eop Kim, Jisu Kim, Sohyun Jeon, Young-Jin Song, Kwang-Ho Choi, Gwangeon Sim, Myunkyu Cho, Jong-Woo Yoon, Hyunsuk Kim

**Affiliations:** Department of Internal Medicine, Hallym University Medical Center, Chuncheon Sacred Heart Hospital, Chuncheon, South Korea

**Keywords:** Autosomal dominant polycystic kidney disease, polycystic liver disease, exercise therapy, bioimpedance

## Abstract

In patients with autosomal dominant polycystic kidney disease (ADPKD) who also have polycystic liver disease (PLD), organomegaly often leads to abdominal symptoms. Abdominal bracing core (ABC) exercises have been validated as effective for alleviating chronic back pain. The purpose of this study was to assess the effectiveness of ABC rehabilitation exercises in reducing pain in ADPKD patients with significant PLD. Significant PLD was defined as a height-adjusted total liver volume (htTLV) exceeding 1,600 mL/m. Both the control groups (*n* = 11) and intervention (*n* = 12) and received an outpatient consultation on nutrition and exercise; however, only the intervention group participated in ABC exercises. After a 3-month biweekly intervention, changes in pain assessed by the Korean Oswestry Disability Index, abdominal symptoms, quality of life (QoL) by the second version of the short-form 36-item Health Survey, and bioelectrical impedance analysis (BIA) were analyzed. The participants comprised 23 individuals (male: 4, female: 19). Their mean age was 54, and the mean ± SD of htTLV was 2,706 ± 1,335 mL/m. The mean ± SD of eGFR was 53.9 ± 29.0 mL/min/1.73 m^2^. After the intervention, pain and pressure-related symptoms significantly decreased in some cases; however, gastrointestinal symptoms did not improve. Pain (control vs. intervention: −1.9 vs. 1.0) and QoL (1.20 vs. 0.93) and showed significant improvements. The results of BIA indicated a noticeable change in the soft lean mass of the proximal body (0.4 vs. −0.2). Our study demonstrates that ABC exercise is effective in alleviating pain and increasing soft lean mass in ADPKD patients who have significant PLD.

## Graphical Abstract

**Figure:**
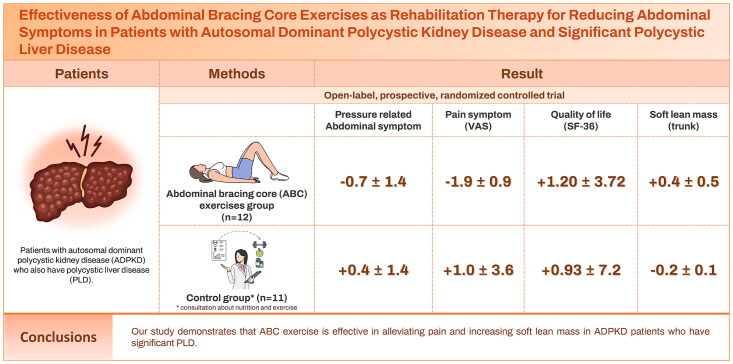

## 1. Introduction

Autosomal dominant polycystic kidney disease (ADPKD) is a systemic disorder characterized by both renal and extra-renal manifestations. It is estimated that approximately 12 million people worldwide have ADPKD, with no significant racial differences in prevalence rates. The prevalence of ADPKD ranges from 1 in 400 to 1 in 1,000. Polycystic liver disease (PLD) is the most common extra-renal manifestation of ADPKD, affecting about 91% of patients with this condition [1].

Although the growth of liver cysts typically does not result in the loss of liver function, the mass effect—physical discomfort caused by bulky cysts—can include abdominal discomfort, dyspepsia, chronic pain, vomiting, difficulty breathing, ascites, and pitting edema. Furthermore, it has been reported that more than 90% of ADPKD patients with PLD, who have a massive liver size exceeding 1,600 mL/m of height-adjusted total liver volume (htTLV), experience chronic back pain and abdominal discomfort [1]. Approximately 3-5% of ADPKD patients experience hepatomegaly-related symptoms [2], which often restrict their mobility due to chronic pain, including back pain, abdominal pain, or discomfort. Additionally, various gastrointestinal symptoms such as dyspepsia, gastroesophageal reflux disease (GERD), loss of appetite, and early satiety hinder their ability to maintain a proper diet. This can exacerbate chronic pain, muscle mass reduction, and malnutrition. However, many patients are reluctant to use painkillers due to concerns about their diminishing renal function, resulting in inadequate pain management.

Bioelectrical impedance analysis (BIA) is a noninvasive tool used to assess body composition and nutritional status, particularly in patients with chronic kidney disease (CKD), including those with end-stage kidney disease (ESKD) [3–6]. Parameters such as intracellular water, extracellular water, total body water, skeletal muscle mass, total fat, and body cell mass (BCM) are measured to evaluate changes in nutrition, physical activity, and disease states [7,8]. BCM, comprising muscle, organ tissue, and blood cells, is a key biomarker for nutritional and metabolic health. In addition to its value for evaluating the nutritional status of CKD patients, BIA serves as a predictive tool for adverse clinical outcomes [9,10]. In patients with ADPKD, BIA has been used to monitor nutritional status, and the leg phase angle has been identified as a relevant parameter. However, the impact of cyst-related water content on BIA measurements in this population remains unclear [11]. Nevertheless, BIA measurements are useful for monitoring the nutritional and physical status of ADPKD patients and observing changes in skeletal muscle mass or BCM after abdominal bracing core (ABC) exercises, assuming no cyst growth in the liver and kidneys for 3 months [11].

ABC exercises [12] are a version of abdominal bracing exercises (McGill, University of Waterloo) [13]. These exercises are widely recognized and have been proven to reduce chronic pain, including back pain [3,14,15]. This method involves education on proper positioning, McKenzie sitting, and intermittent abdominal bracing. Although evidence specific to ADPKD patients is limited, it is well-established that strengthening core muscles is effective for managing back pain [16]. The high prevalence of pain in ADPKD patients with PLD [1] has prompted the hypothesis that insufficient core muscle mass could contribute to this symptom. Therefore, targeted interventions to increase core muscle mass through exercise may help alleviate pain in this patient population.

This study hypothesized that repetitive ABC exercises can reduce pain in the back, abdomen, and flanks of ADPKD patients with PLD. By alleviating pain and improving exercise tolerance, this intervention may also mitigate the mass effect of hepatomegaly, improve quality of life (QoL), and increase muscle mass. We aimed to evaluate the effects of ABC exercises on abdominal symptoms, QoL, and body composition in ADPKD patients with PLD.

## 2. Materials and methods

### 2.1. Patients

This study was designed as an open-label, prospective, randomized controlled trial to evaluate the potential of ABC exercises as a new effective treatment. We collected clinical data over a four-month period (from November 2014 to February 2015) from 23 ADPKD patients, aged 37 to 67, at the ADPKD clinic of Seoul National University Hospital. The study design was informed by a previous study that measured the average visual analog scale (VAS) change following abdominal stabilizing exercises in a randomized control trial [17]. The participants were divided into two groups: an intervention group of 12 patients who engaged in ABC exercises six times over 3 months (once every 2 weeks), and a control group of 11 patients who received only nutritional consultations and recommendations for regular exercise. In our study, one attending physician provided tailored guidance on low-sodium diets, protein intake, and caloric needs according to each patient’s CKD stage. Additionally, we provided ­education to exercise in accordance with the 2010 WHO recommendations, which include at least 150 min of moderate-intensity aerobic activity or 75 min of vigorous-intensity ­aerobic activity per week, along with muscle-strengthening activities at least twice a week. The study was conducted in accordance with the Declaration of Helsinki, and approved by the Institutional Review Board of Seoul National University Hospital (H-1002-028-311). All patients provided written informed consent prior to participation.

All 23 patients were selected because they satisfied the unified ultrasonographic criteria for ADPKD1 (3 or more unilateral or bilateral cysts in age 15-39, 2 or more cysts in each kidney in age 40-59, 4 more cysts in each kidney in age 60 or more) [18] and had significant PLD, defined by an htTLV exceeding 1,600 mL/m [1]. Total liver volume (TLV) was measured using stereologic volumetry with the Rapidia program (version 2.8; Infinitt Co. Ltd., Seoul, Korea). Additionally, all patients had experienced chronic pain in the back, flank, or abdomen for more than three months, which they rated as 2 or higher on the VAS. Patients who were already being treated for back, flank, or abdominal pain and those being treated for progressive cancer by chemotherapy or radiotherapy were excluded from the patient group in advance. Before and after the ABC exercises, all patients were assessed for ADPKD-related abdominal symptoms using a premade ­questionnaire from a previously published paper. This questionnaire included 11 items on pressure, pain, and gastrointestinal-related symptoms [2]. Data were also collected on the Korean Oswestry Disability Index (KODI), the second version of the short-form 36-item Health Survey (SF-36 v2), and body impedance analysis (BIA) using Inbody (S12, Seoul, Korea). We ensured the absence of edema before conducting BIA measurements. The formula provided by InBody applies the Cunningham method to calculate basal metabolic rate (BMR = Ideal fat free mass*21.6 + 370) based on the ideal lean body mass derived from the current body weight [19]. The KODI is used to measure functional disability due to low back pain, and the SF-36 v2 assesses QoL [20,21].

### 2.2. ABC exercises

The ABC exercise program for ADPKD patients was conducted in two phases; stage 2 was planned to start after patients were accustomed to stage 1 exercises, since the exercises in stage 2 comprised more complicated postures that were harder to follow, but beneficial to augment the effects of the ABC exercises. Only the patients in the intervention group participated in biweekly 30- to 40-min exercise sessions. A specialist in rehabilitation medicine participated in providing training, and one trained nephrology healthcare professional assisted in ensuring that patients maintained a proper posture during the exercises. The detailed exercise protocols can be found in the Data S^1^.

In stage 1, seven procedures were implemented: McKenzie extension exercise, abdominal bracing (three times a day), sitting posture, standing posture, abdominal bracing with deep breathing (three sets a day, 10 repetitions per set, performed in either sitting or standing posture), sitting to standing, and abdominal bracing with walking. During the McKenzie extension exercise, patients are required to arch their torso backwards and hold this position for 5 s. Abdominal bracing involves pushing the abdomen and pelvis forward to relax the back muscles. The sitting posture entails slightly tightening the abdomen while sitting on a chair with legs apart. Conversely, the standing posture involves tightening the abdomen while maintaining an upright stance. Abdominal bracing with deep breathing consists of taking a deep breath while relaxing the back muscles by pushing the abdomen and pelvis forward. The sitting to standing exercise requires tightening the abdomen when transitioning from a sitting to a standing position, using the strength of the knees. Lastly, abdominal bracing with walking involves walking in a straight line while engaging the abdominal muscles.

In stage 2, there were also seven procedures: hip hinge – kneeling posture, potty squat, curl-up (beginner), bridge exercise for gluteus maximus, bird-dog (beginner), knee clam exercise for gluteus maximus, and side bridge exercise. Each procedure in stage 2 was performed in three sets. The hip hinge – kneeling posture involves pushing the hips backward while maintaining an abdomen braced in a kneeling position. The potty squat entails performing a squat while bracing the abdomen and buttocks. The curl-up (beginner version) involves bending the knees and lifting the torso while lying on the back. The bridge exercise focuses on tightening the buttocks and lifting the pelvis while the knees are bent and the individual is lying down. The bird-dog (beginner version) requires lifting one hand or knee in a dog posture. The knee clam exercise involves lifting the upper knee, mimicking a clam, while lying on one side. The side bridge exercise involves lifting the pelvis from the floor while lying on one side with the knees bent at 45°; this exercise should be performed in three consecutive sets. Since all patients successfully completed the stage 1 exercises, we proceeded with stage 2 exercises starting from the third meeting (Data S^1^).

### 2.3. Statistical analysis

All statistics—including the baseline characteristics of the 23 patients, baseline symptom scores before ABC exercises, and changes in body composition between both groups—are presented as mean ± standard deviation (SD). Each statistic was analyzed using the paired t-test to calculate the p-value with SPSS ver. 23.0. The randomized division of patients into two groups is evidenced by p-values greater than 0.05 for related statistics. Additionally, we visualized the outcomes using line and bar graphs.

## 3. Results

### 3.1. Baseline demographic characteristics of the intervention and control groups

The total number of ADPKD patients with significant PLD was 23, divided into two groups as previously mentioned: 11 in the control group and 12 in the intervention group. Of these, 20 patients (87%) were female, with 9 (82%) in the control group and 11 (92%) in the intervention group. The average age of all patients was 54 ± 9 years, with those in the control group averaging 51 ± 9 years and intervention group averaging 57 ± 9. The htTLV for all patients was 2,706 ± 1,335 mL/m, with values of 2,697 ± 1,393 mL/m in the control group and 2713 ± 1,343 mL/m in the intervention group. Creatinine levels averaged 1.7 ± 1.4 across all patients, with levels of 1.7 ± 1.6 in the control group and 1.7 ± 1.3 in the intervention group. Similarly, the estimated glomerular filtration rate (eGFR) was 55.4 ± 29.0 mL/min/1.73 m^2^ for all patients, with values of 49.6 ± 25.7 mL/min/1.73 m^2^ in the control group and 61.2 ± 32.2 mL/min/1.73 m^2^ in the intervention group. Two patients, both in the intervention group, received renal replacement therapy: one underwent hemodialysis, and the other received kidney transplantation. Overall, the patients in the intervention group tended to be older, but there was no significant age difference between the two groups (Table 1).

**Table 1.** Baseline characteristics of the control and intervention groups.

	Total	Control	Intervention	*P*-value
n	23	11	12	
Female, n (%)	20 (87)	9 (82)	11 (92)	0.590
Age, mean ± SD (years)	54 ± 9	51 ± 9	57 ± 9	0.091
htTLV, mean ± SD (mL/m)	2,706 ± 1335	2,697 ± 1393	2,713 ± 1343	0.976
Creatinine, mean ± SD (mg/dL)	1.7 ± 1.4	1.7 ± 1.6	1.7 ± 1.3	0.562
eGFR (mL/min/1.73 m^2^)	55.4 ± 29.0	49.6 ± 25.7	61.2 ± 32.2	0.786
RRT, n	2	0	2, 1 HD 1 KT	0.478

SD: standard deviation; htTLV: height-adjusted total liver volume; eGFR: estimated glomerular filtration rate; RRT: renal replacement therapy; HD: hemodialysis; KT: kidney transplantation.

### 3.2. Baseline symptom scores in the intervention and control groups

Baseline symptom scores were measured in 23 patients using four methods: ADPKD abdominal symptoms, KODI, SF-36 v2 – physical health, and SF-36 v2 – mental health. The results are presented as [control group vs. intervention group]. First, ADPKD abdominal symptoms were evaluated in three aspects: pressure-related, pain, and gastrointestinal. The pressure-related score was 2.6 ± 1.3 vs. 3.0 ± 2.0. The pain score was 2.9 ± 0.9 vs. 3.2 ± 1.6. The gastrointestinal score was 1.7 ± 0.9 vs. 1.6 ± 1.3. Second, the KODI was assessed in terms of the pain VAS and pain-related QoL. The pain VAS score was 3.4 ± 1.7 vs. 4.1 ± 1.6. The pain-related QoL score was 15.7 ± 11.8 vs. 22.3 ± 12.1. Third, SF-36 v2 – physical health was evaluated across five aspects: physical component summary (PCS), general health, physical functioning, role limitations due to physical problems, and bodily pain. The PCS score was 43.3 ± 5.4 vs. 45.6 ± 5.6. Lastly, SF-36 v2 – mental health was also evaluated in five aspects: mental component summary (MCS), mental health, vitality, social functioning, and role limitations due to emotional problems. The MCS score was 45.7 ± 6.6 vs. 48.0 ± 9.3. In summary, abdominal symptoms, KODI pain scores, and SF-36 scores were not meaningfully different between the two groups (Table S1).

### 3.3. Body composition parameters in the intervention and control groups

Body composition measurements, including body mass index (BMI), protein, fat, soft lean mass, skeletal muscle mass, body cell mass, arm muscle circumference, visceral fat, and basal metabolic rate, were obtained for both the control and intervention groups. The results, presented as mean ± standard deviation (SD) [control group vs. intervention group], showed no significant differences between the baseline measurements of the control and intervention groups (Table S2).

### 3.4. Changes in abdominal symptoms after ABC exercises

After three months of ABC exercises in the intervention group, we re-measured ADPKD abdominal symptoms, KODI, SF-36 v2, and BIA parameters to compare them within and between the control and intervention groups, as described in greater detail below. Before the re-measurement of ADPKD abdominal symptoms, one patient in the intervention group and five in the control group dropped out, leaving 11 and 6 patients, respectively.

Regarding the pressure-related symptoms score, in the intervention group, scores decreased for five patients, remained the same for four, and increased for two. In the control group, scores decreased for one patient, remained the same for three, and increased for two. The net average change in pressure-related symptoms scores was +0.4 ± 1.4 for the control group vs −0.7 ± 1.4 for the intervention group (p-value within the control group = 0.448, p-value within the intervention group = 0.120, p-value between the two groups = 0.170).

For the pain score, in the intervention group, four patients reported decreased scores, seven remained the same, and none increased. In the control group, scores decreased for two patients, remained the same for two, and increased for two. The net average change in pain scores was −0.1 ± 1.8 for the control group vs −0.5 ± 0.9 for the intervention group (*p* = 0.838, 0.082, 0.942).

For the GI symptom score, in the intervention group, scores decreased for four patients, remained the same for three, and increased for four. In the control group, scores decreased for four patients, remained the same for two, and none increased. The net average change in the GI symptom scores was −0.7 ± 0.8 for the control group vs −0.2 ± 1.2 for the intervention group (*p* = 0.047, 0.617, 0.184).

**Figure 1.**
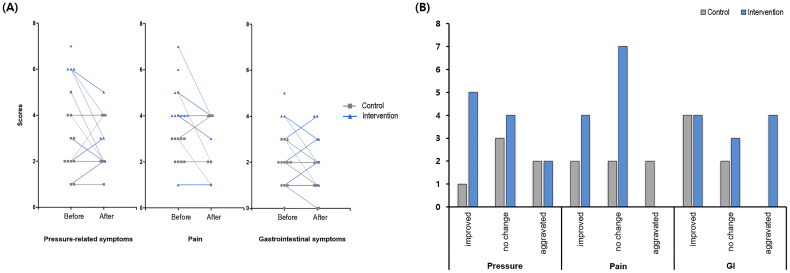
Changes in abdominal symptoms in the control and intervention groups: (A) changes of scores between 2 groups in pressure, pain, and GI symptoms, (B) symptom change distribution: Improved, No change, and aggravated in pressure, pain, and GI symptoms.

The results suggest that ABC exercises may be relatively effective in alleviating abdominal symptoms and pain in the intervention group. However, the p-values between the two groups were not significant (Figure 1).

### 3.5. Changes in KODI scores after ABC exercises

Before re-measuring the KODI score, one patient in the intervention group and six patients in the control group dropped out, leaving 11 and 6 patients remaining in each group, respectively. As shown in Figure S1, regarding the pain VAS score, in the intervention group, 11 patients experienced a decrease, while none remained the same or showed an increase. In the control group, two patients reported a decrease in pain, none remained the same, and three experienced an increase. The net average change in the pain VAS score was +1.0 ± 3.6 for the control group vs −1.9 ± 0.9 for the intervention group (*p* = 0.524, <0.001, <0.001).

For the pain-related QoL score, in the intervention group, nine patients showed a decrease, two remained the same, and none increased. In the control group, no patients reported a decrease, one remained the same, and four showed an increase. The net average change in the pain-related QoL score was +7.9 ± 8.8 for the control group vs −10.0 ± 12.7 for the intervention group (*p* = 0.055, 0.020, 0.774). Pain-related scores demonstrated a significant reduction in the intervention group compared to the control group. However, the p-value for the pain-related QoL between the two groups was not significant.

### 3.6. Changes in QoL measured by the SF-36 v2 score after ABC exercises

**Figure 2.**
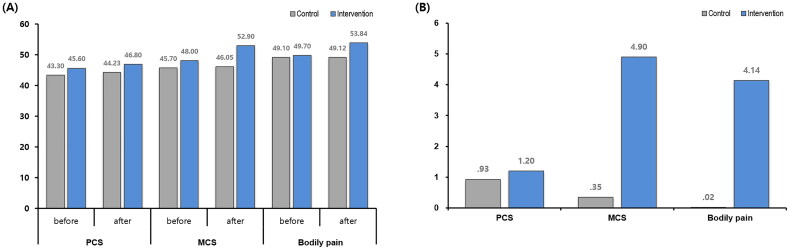
Changes in quality of life (SF-36 v2) in the control and intervention groups: (A) scores of PCS, MCS, and bodily pain before and after between 2 groups, (B) changes of scores of PCS, MCS, and bodily pain between 2 groups. PCS: physical component summary; MCS: mental component summary.

This bar graph compared the scores of the control group and the intervention group across three domains PCS, MCS, and Bodily Pain, measured before and after the intervention. In PCS, both groups showed slight improvements, with scores increasing from 43.30 to 44.23 in the control group and from 45.60 to 46.80 in the intervention group. For MCS, the control group’s score rose modestly from 45.70 to 46.05, while the intervention group showed a more substantial improvement from 48.00 to 52.90. Similarly, in Bodily Pain, the control group remained almost unchanged (49.10 to 49.12), whereas the intervention group experienced a noticeable increase from 49.70 to 53.84 (Figure 2A). A difference in QoL measured by the SF-36 v2 was identified between the control and intervention groups. For the PCS, the average change in QoL was +0.93 ± 7.2 (*p* = 0.854) in control group, whereas it was +1.20 ± 3.72 (*p* = 0.310) in the intervention group. There was a significant difference for MCS; the average score change was +0.35 ± 6.55 (*p* = 0.310) in the control group and +4.90 ± 8.05 (*p* = 0.071) in the intervention group. The disparity was even more pronounced in bodily pain, with the average change in QoL being 0.02 ± 8.74 (*p* = 0.755) in the control group and 4.14 ± 6.16 (*p* = 0.050) in the intervention group. However, there were no significant differences between the two groups (Figure 2B).

### 3.7. Changes in body composition after ABC exercises

Changes in body composition were measured in both the control and intervention groups, with results presented as mean ± standard deviation (SD) for the intervention group vs. control group. The changes in BMI were 0.1 ± 0.6 vs. −0.6 ± 0.3 (*p* = 0.63), those in total body protein levels were 0.1 ± 0.4 vs. 0.2 ± 0.4 (*p* = 0.143), and those in total fat levels were 0.7 ± 1.7 vs. −0.5 ± 1.3 (*p* = 0.124). For soft lean mass in the right arm, the changes were −0.1 ± 0.1 vs. 0.1 ± 0.1 (*p* = 0.003). In the left arm, the corresponding changes were −0.1 ± 0.0 vs. 0.1 ± 0.1 (*p* = 0.001). Significant changes were observed in the body trunk: −0.2 ± 0.1 vs. 0.4 ± 0.5 (*p* = 0.021). In the right leg, the changes were −0.1 ± 0.3 vs. 0.1 ± 0.3 (*p* = 0.577). In the left leg, the changes were −0.1 ± 0.2 vs. 0.3 ± 0.3 (*p* = 0.512). The changes in skeletal muscle mass were −0.5 ± 0.9 vs. 0.5 ± 1.2 (*p* = 0.148), those in body cell mass were −0.5 ± 1.0 vs. 0.3 ± 1.3 (*p* = 0.276), and those in arm muscle circumference were −0.1 ± 0.2 vs. 0.3 ± 0.5 (*p* = 0.024). The changes in visceral fat area were 2.0 ± 9.1 vs. −3.5 ± 8.9 (*p* = 0.284). The change in the basal metabolic rate was −19.6 ± 30.0 vs. 15.3 ± 44.0 (*p* = 0.097). Consequently, the most notable differences in body composition changes were primarily observed in the soft lean mass of the arms and trunk (Table 2, Figure S2).

**Table 2.** Changes in body composition in the control and intervention groups.

	Control	Intervention	*P*-value
BMI, kg/m2, mean ± SD	0.1 ± 0.6	−0.6 ± 0.3	0.630
Total protein, kg, mean ± SD	0.1 ± 0.4	0.2 ± 0.4	0.143
Total fat, kg, mean ± SD	0.7 ± 1.7	−0.5 ± 1.3	0.124
Soft lean mass, kg, mean ± SD			
Right arm	−0.1 ± 0.1	0.1 ± 0.1	0.003
Left arm	−0.1 ± 0.0	0.1 ± 0.1	0.001
Trunk	−0.2 ± 0.1	0.4 ± 0.5	0.021
Right leg	−0.1 ± 0.3	0.1 ± 0.3	0.577
Left leg	−0.1 ± 0.2	0.3 ± 0.3	0.512
Skeletal muscle mass, kg, mean ± SD	−0.5 ± 0.9	0.5 ± 1.2	0.148
Body cell mass, kg, mean ± SD	−0.5 ± 1.0	0.3 ± 1.3	0.276
Arm muscle circumference, kg, mean ± SD	−0.1 ± 0.2	0.3 ± 0.5	0.024
Visceral fat area, kg, mean ± SD	2.0 ± 9.1	−3.5 ± 8.9	0.284
Basal metabolic rate, kg, mean ± SD	−19.6 ± 30.0	15.3 ± 44.0	0.097

BMI: body mass index; SD: standard deviation.

### 3.8. Discussion

Hepatomegaly, which results from PLD combined with ADPKD, can lead to malnutrition in patients. This condition causes abdominal distention, early satiety, postprandial fullness, GERD, dyspnea, and back pain, all of which can impair a patient’s ability to maintain an adequate nutritional status [1]. A study assessing nutritional status using the subjective global assessment (SGA) in ADPKD patients with hepatomegaly revealed that patients with height-adjusted total kidney and liver volume values exceeding 2340 mL/m had an eightfold increased risk of malnutrition (SGA scores of 4 and 5) [22].

Approximately 45-70% of ADPKD patients progress to ESKD by the age of 65 years [23]. Malnutrition worsens the prognosis of ESKD patients, as it is associated with increased risks of mortality, the composite endpoint of death or dialysis, and acute hospitalization [24]. Hence, it is generally recommended to administer protein as nutritional support on a daily basis for ESKD patients [25]. However, protein nutritional support does not address muscle wasting, which also contributes to increased dependency, mortality, and morbidity in CKD patients [26]. It is uncertain whether supportive management of protein intake actually mitigates muscle wasting, as the direct relationship between the two has not been established. Instead, muscle wasting is thought to result from factors such as protein synthesis, degradation, and inactivity [27]. Although both inappropriate protein intake and muscle loss are predictors of poor prognosis in CKD, the primary treatment focus for malnutrition centers on the quantity of protein administered to the patient. This highlights the need to consider physical treatment options, such as exercise, in the management strategy.

Pain is one of the most common symptoms of ADPKD, with reports indicating that 71% of patients experience low back pain. The causes of pain in ADPKD vary and include cyst hemorrhage, urinary tract infections, pyelonephritis, and nephrolithiasis for acute pain; pressure from enlarged cysts and ongoing pain from sensitization even after the causal cyst has been removed for chronic pain. Additionally, pain can originate from the liver due to heaviness, dull ache, mechanical pressure, and vessel compression [28]. Some patients also develop lumbar lordosis due to hypertrophy of the lumbodorsal muscle and disk disease resulting from pelvic shift [29]. To manage this pain, most treatments focus on medications and surgical interventions such as analgesia, tramadol, transcutaneous electric nerve stimulation, cyst aspiration, renal denervation, and nephrectomy. However, only a few noninvasive techniques like ice massage, heating, and the Alexander technique are recommended for non-pharmacologic and physical measures [30]. In this context, a pain-control method through exercise, which could provide a continuous and straightforward way to manage pain, would be a novel approach for ADPKD patients experiencing chronic pain.

Due to factors including renal insufficiency in CKD and ADPKD, maintaining proper nutrition through diet alone is challenging. Additionally, pain management in ADPKD patients typically involves painkillers, which do not address the underlying cause of the pain. In such cases, an exercise regimen like the ABC exercise could serve as an alternative and supportive treatment for patients with hepatomegaly associated with ADPKD. Research has been conducted on the impact of exercise on CKD patients to determine whether it improves their physical function or overall health status [31–33]. Although there is currently no established exercise protocol for CKD [34,35], evidence suggests that exercise generally has a positive effect on the prognosis and quality of life for these patients [36,37].

Given the lack of specific exercise intervention programs for CKD patients, this study is pioneering in proposing a specialized exercise method designed for ADPKD patients with PLD who suffer from chronic pain. As demonstrated, ABC exercises for ADPKD patients can improve the BIA parameter, particularly the soft lean mass in the upper body; it can also alleviate pain and pressure-related symptoms, potentially leading to an enhanced quality of life for these individuals. For soft lean mass changes in the upper body, even though the ABC exercises mainly comprised trunk exercises, increased arm strength would have been a positive side effect. Some of the stage 2 exercises need arm strength to maintain the posture and the statistical results regarding the high proportion of soft lean mass in the arms were less promising than those for the trunk. Furthermore, although this study aimed to achieve pain reduction in patients with ADPKD and PLD, other previous studies have shown that exercise can yield effective results for pain modulation by lumbopelvic core stabilization [38]. More than 50% of ADPKD patients report experiencing back and abdominal pain due to mass effects resulting from enlargement of the kidneys and liver. Therefore, the ABC exercises are likely to be beneficial for ADPKD patients with significantly enlarged polycystic kidneys and polycystic liver.

### 3.9. Limitations

This study has several limitations, most of which originated from the relative rarity of ADPKD. Primarily, the sample size of the study was small and the dropout rate was higher than expected in the control group, which might have caused unexpected statistical bias. However, considering the overall consistency regarding changes after the intervention and the need for supportive management other than medications for ADPKD patients, future large population-based cohort studies would be recommended for higher generalizability.

Moreover, as there is no firmly established or frequently used tool to assess the nutritional or muscular status of ADPKD patients, BIA might not be the best option for evaluating ADPKD patients since it is known to be influenced by fluid status, and ADPKD patients generally have a higher fluid composition because of their renal and hepatic cysts. Despite the potential inaccuracy of these measurements, the results regarding changes in status are meaningful based on the assumption that a patient’s fluid status would not dramatically change in 3 months. Nonetheless, another suggestion for future research would be to utilize a more recently authorized tool to increase the precision of the study.

## 4. Conclusion

In this study, we demonstrated that ABC exercises effectively relieved pain and improved pain-related QoL measures (PCS, MCS, bodily pain) in ADPKD patients with significant PLD. However, there were no significant differences in pressure-related and gastrointestinal symptoms or QoL between the two groups. The results from BIA showed that the soft tissue lean mass in the arms and trunk increased significantly after the ABC exercise regimen. Given the limited research on the effectiveness of ABC exercise in ADPKD patients with hepatomegaly, further studies are necessary to establish ABC exercise as a clinically recognized treatment for ADPKD with PLD.

## Supplementary Material

F1.tif

Supplementary_Tables_241219 new.docx

Supplementary_Figures_241219 new.docx

F2.tif

Supplementary data1.pptx

GraphicalAbstract1.tif

## Data Availability

The data that support the findings of this study are available from the corresponding author, HK, upon reasonable request.
