# Supplementary figures and images for: Effectiveness of abdominal bracing core exercises as rehabilitation therapy for reducing abdominal symptoms in patients with autosomal dominant polycystic kidney disease and significant polycystic liver disease
Source: Ren Fail. 2025 Mar 11;47(1):2457519. doi: 10.1080/0886022X.2025.2457519 (PMC11905316; doi:10.1080/0886022X.2025.2457519)

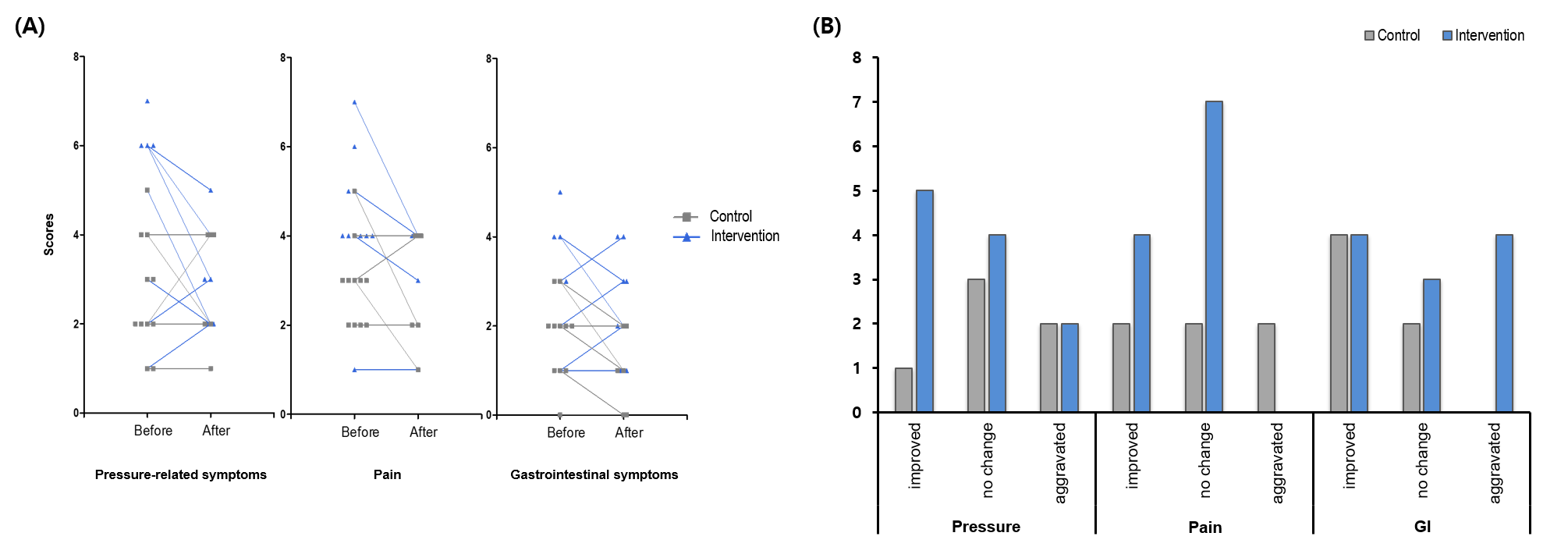

Supplement: F1.tif [file IRNF_A_2457519_SM0800.tif]

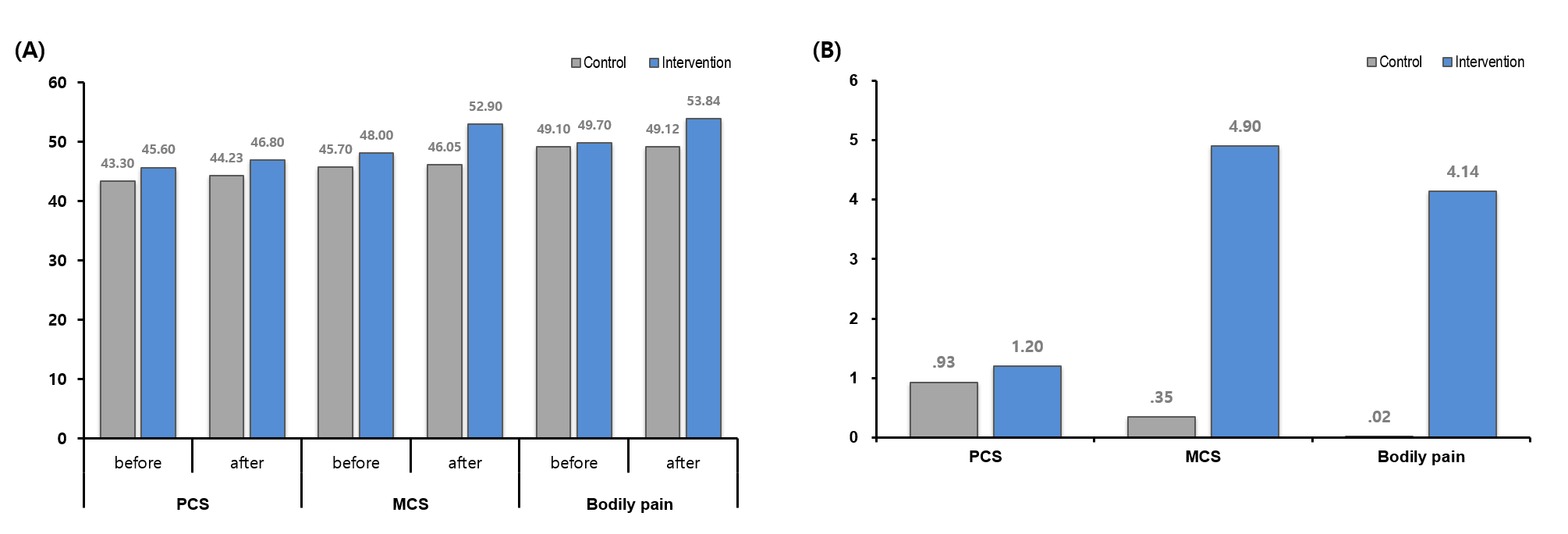

Supplement: F2.tif [file IRNF_A_2457519_SM0797.tif]

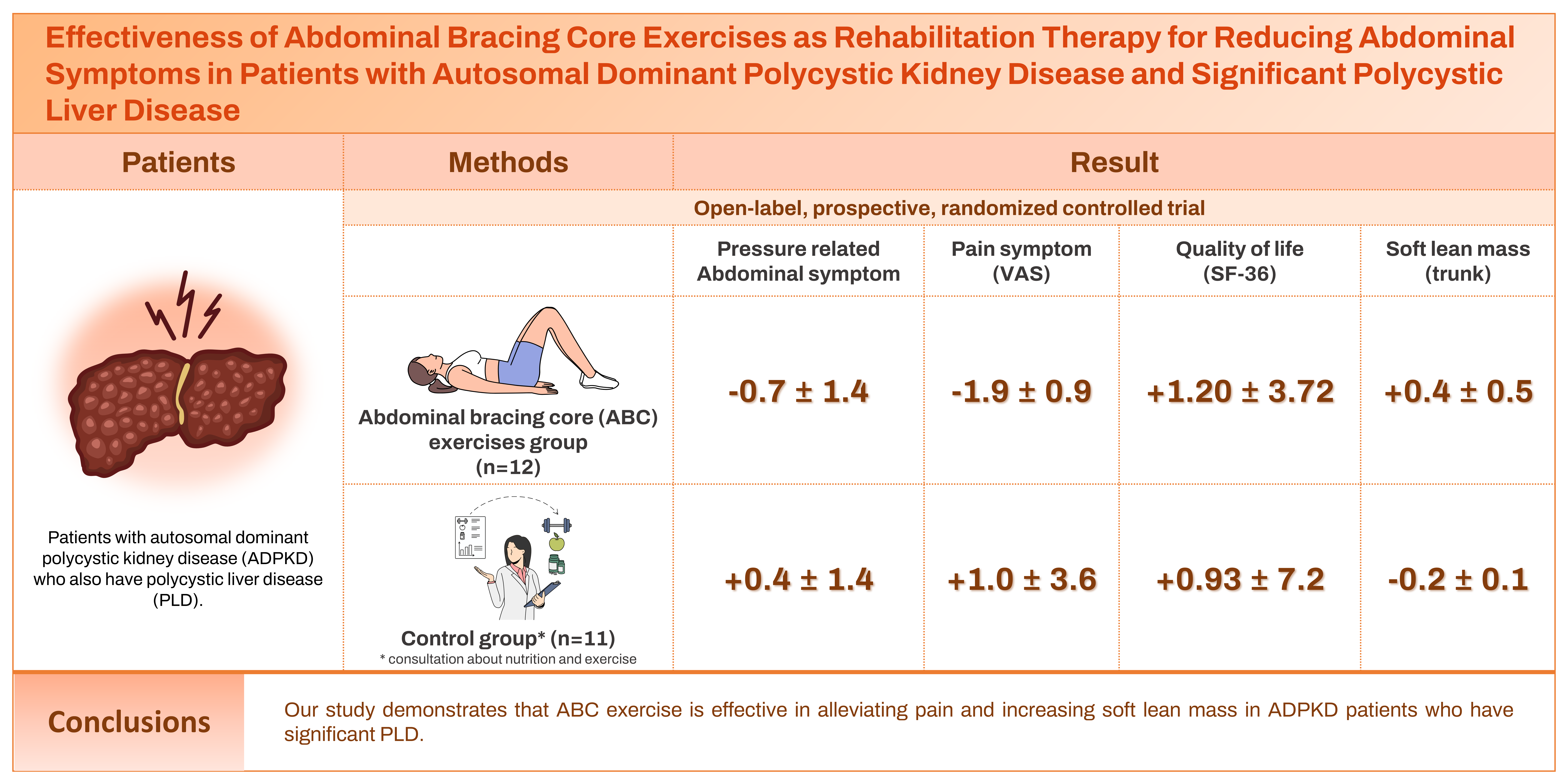

Supplement: GraphicalAbstract1.tif [file IRNF_A_2457519_SM0795.tif]
