# Supplementary material for: Effectiveness of abdominal bracing core exercises as rehabilitation therapy for reducing abdominal symptoms in patients with autosomal dominant polycystic kidney disease and significant polycystic liver disease
Source: Ren Fail. 2025 Mar 11;47(1):2457519. doi: 10.1080/0886022X.2025.2457519 (PMC11905316; doi:10.1080/0886022X.2025.2457519)
Supplement: Supplementary_Tables_241219 new.docx [file IRNF_A_2457519_SM0799.docx]

**Supplementary Table 1.** Baseline symptom scores before ABC exercises in the control and intervention groups

|  | Total | Control | Intervention | P value |
| --- | --- | --- | --- | --- |
| n | 23 | 11 | 12 |  |
| Abdominal symptoms |  |  |  |  |
| Pressure-related, mean ± SD | 2.8 ± 1.7 | 2.6 ± 1.3 | 3.0 ± 2.0 | 0.613 |
| Pain, mean ± SD | 3.0 ± 1.3 | 2.9 ± 0.9 | 3.2 ± 1.6 | 0.645 |
| Gastrointestinal, mean ± SD | 1.7 ± 1.1 | 1.7 ± 0.9 | 1.6 ± 1.3 | 0.764 |
| KODI |  |  |  |  |
| Pain_VAS, mean ± SD | 3.7 ±1.6 | 3.4 ± 1.7 | 4.1 ± 1.6 | 0.301 |
| Pain related QoL, mean ± SD | 19.1 ± 12.2 | 15.7 ± 11.8 | 22.3 ± 12.1 | 0.194 |
| SF-36v2 |  |  |  |  |
| Physical health |  |  |  |  |
| Physical component summary, mean ± SD | 44.4 ± 6.3 | 43.3 ± 5.4 | 45.6 ± 5.6 | 0.375 |
| General health | 36.1 ± 9.1 | 33.4 ± 5.9 | 38.6 ± 11.0 | 0.177 |
| Physical functioning, mean ± SD | 44.0 ± 8.3 | 44.3 ± 10.1 | 43.7 ± 6.8 | 0.858 |
| Role limitations due to physical problems, mean ± SD | 45.7 ±9.2 | 45.1 ± 10.3 | 46.3 ± 8.5 | 0.766 |
| Bodily pain | 49.4 ±8.1 | 49.1 ± 8.0 | 49.7 ± 8.5 | 0.869 |
| Mental health |  |  |  |  |
| Mental component summary, mean ± SD | 46.9 ± 8.0 | 45.7 ± 6.6 | 48.0 ± 9.3 | 0.516 |
| Mental health, mean ± SD | 46.7 ± 10.2 | 47.5 ± 7.3 | 46.1 ± 12.6 | 0.750 |
| Vitality, mean ± SD | 43.3 ± 5.4 | 43.3 ± 5.4 | 50.5 ± 12.1 | 0.080 |
| Social functioning, mean ± SD | 47.9 ± 7.4 | 48.7 ± 9.5 | 48.7 ± 9.5 | 0.838 |
| Role limitations due to emotional problems, mean ± SD | 42.8 ± 6.6 | 47.6 ± 12.6 | 47.6 ± 12.6 | 0.339 |

*Abbreviations*: SD, standard deviation; KODI, Korean Oswestry Disability Index; VAS, visual analog scale

**Supplementary Table 2.** Baseline body composition of the control and intervention groups

|  | Control | Intervention | P value |
| --- | --- | --- | --- |
| n | 11 | 12 |  |
| BMI, kg/m2, mean ± SD | 23.5 ± 2.5 | 21.8 ± 2.7 | 0.151 |
| Total protein, kg, mean ± SD | 9.5 ± 1.2 | 12.2 ± 11.7 | 0.485 |
| Total fat, kg, mean ± SD | 13.2 ± 5.5 | 13.3 ± 4.7 | 0.946 |
| Soft lean mass, kg, mean ± SD |  |  |  |
| Right arm | 2.4 ± 0.4 | 2.6 ± 1.5 | 0.711 |
| Left arm | 2.4 ± 0.4 | 4.6 ± 8.1 | 0.412 |
| Trunk | 20.3 ± 2.4 | 17.8 ± 5.3 | 0.183 |
| Right leg | 8.1 ± 4.4 | 8.7 ± 6.6 | 0.554 |
| Left leg | 7.5 ± 1.3 | 8.1 ± 4.4 | 0.680 |
| Skeletal muscle mass, kg, mean ± SD | 26.1 ± 4.9 | 21.9 ± 8.2 | 0.326 |
| Body cell mass, kg, mean ± SD | 31.5 ± 3.9 | 32.5 ± 12.3 | 0.799 |
| Arm muscle circumference, cm, mean ± SD | 23.1 ± 1.5 | 21.5 ± 4.3 | 0.280 |
| Visceral fat area, cm2, mean ± SD | 57.7 ± 22.6 | 52.5 ± 20.1 | 0.573 |
| Basal metabolic rate, kcal, mean ± SD | 1439.7 ± 140.9 | 1252.2 ± 415.6 | 0.189 |

*Abbreviations*: BMI, body mass index; SD, standard deviation
