# Supplementary material for: Effectiveness of abdominal bracing core exercises as rehabilitation therapy for reducing abdominal symptoms in patients with autosomal dominant polycystic kidney disease and significant polycystic liver disease
Source: Ren Fail. 2025 Mar 11;47(1):2457519. doi: 10.1080/0886022X.2025.2457519 (PMC11905316; doi:10.1080/0886022X.2025.2457519)
Supplement: Supplementary_Figures_241219 new.docx [file IRNF_A_2457519_SM0798.docx]

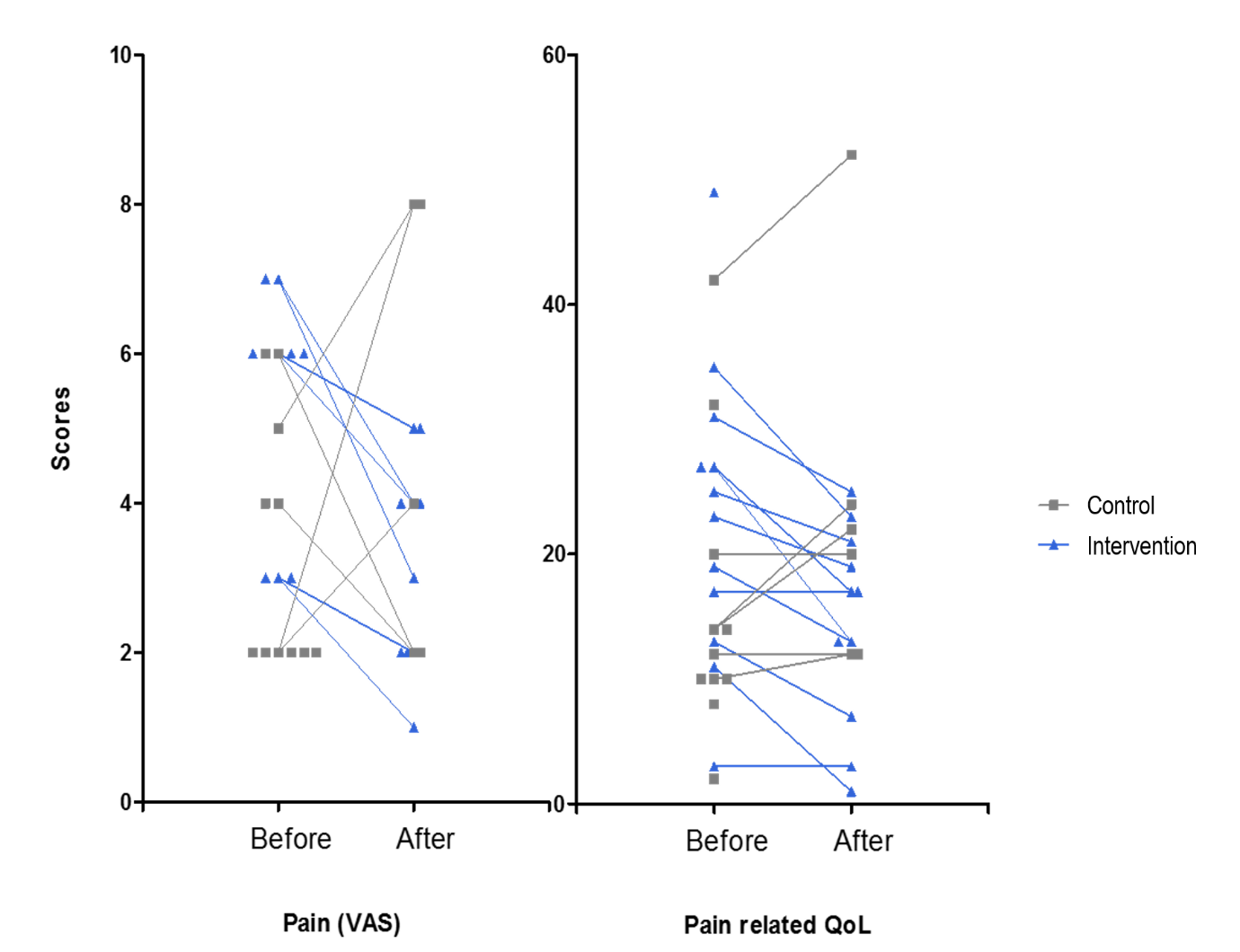


**Supplementary Fig. 1** Changes in pain scores (KODI) in the control and intervention groups


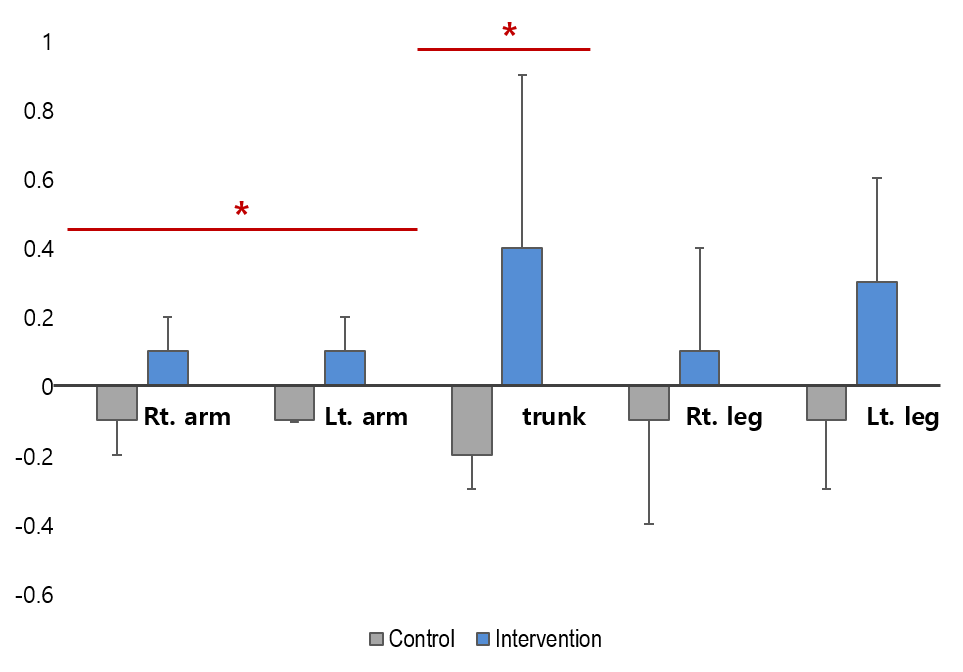


**Supplementary Fig. 2** Changes in Inbody muscle parameters in the control and intervention groups
