# Supplementary material for: Effectiveness of abdominal bracing core exercises as rehabilitation therapy for reducing abdominal symptoms in patients with autosomal dominant polycystic kidney disease and significant polycystic liver disease
Source: Ren Fail. 2025 Mar 11;47(1):2457519. doi: 10.1080/0886022X.2025.2457519 (PMC11905316; doi:10.1080/0886022X.2025.2457519)
Supplement: Supplementary data1.pptx [file IRNF_A_2457519_SM0796.pptx]

## Slide 1
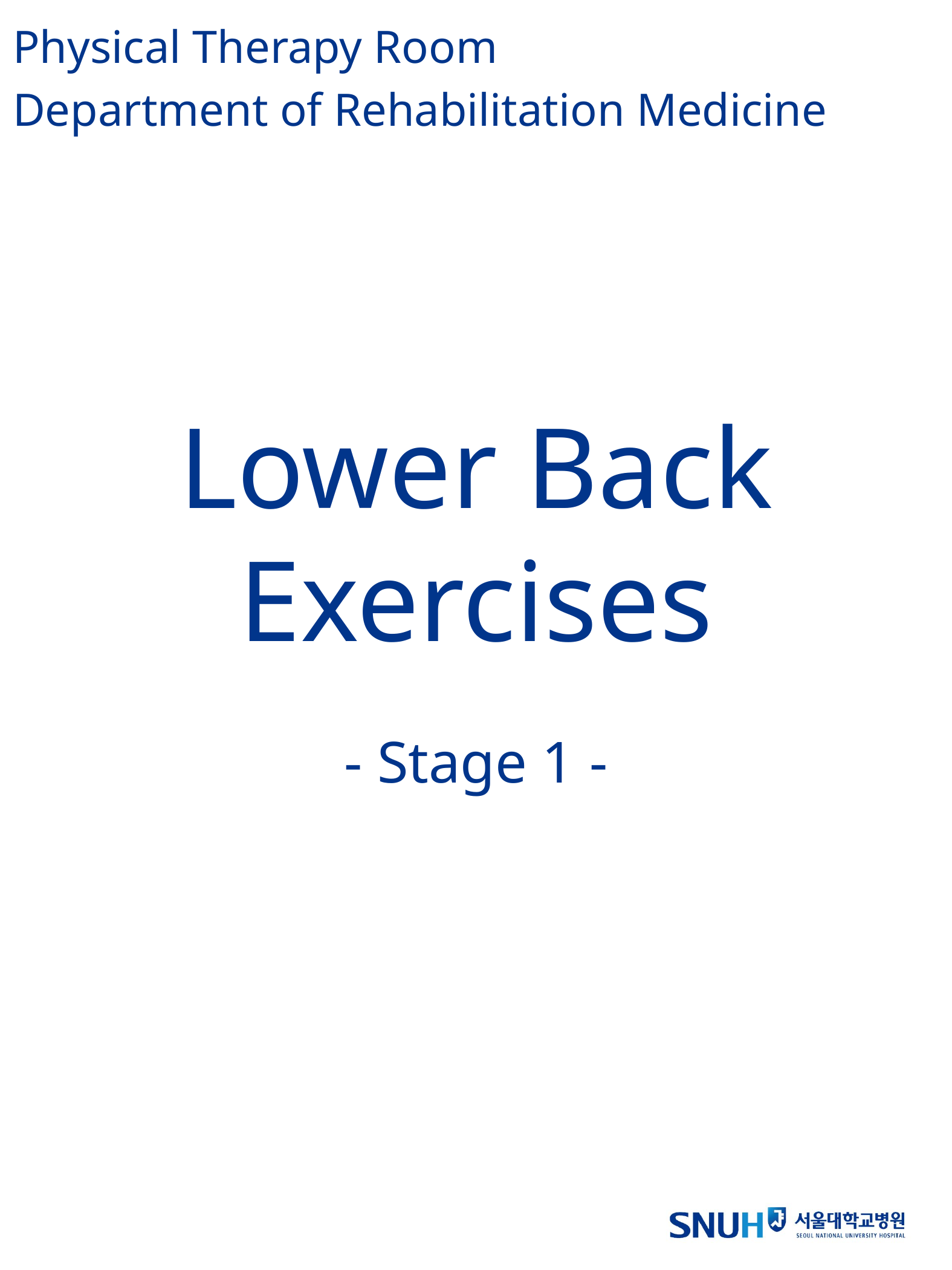

Physical Therapy Room
Department of Rehabilitation Medicine
# Lower Back Exercises
- Stage 1 -

## Slide 2
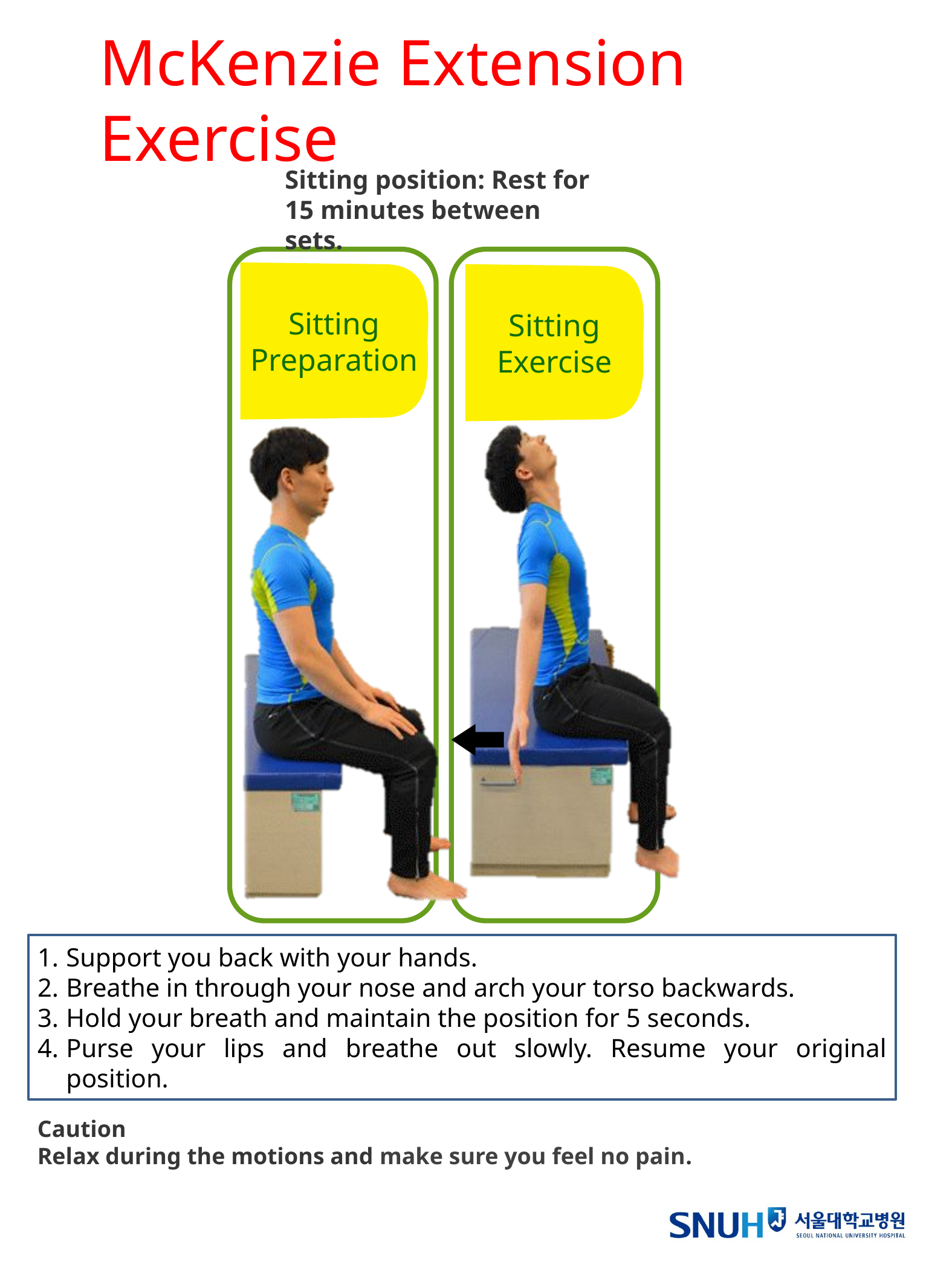

McKenzie Extension Exercise
Sitting position: Rest for
15 minutes between sets.
Sitting
Preparation
Sitting
Exercise
Support you back with your hands.
Breathe in through your nose and arch your torso backwards.
Hold your breath and maintain the position for 5 seconds.
Purse your lips and breathe out slowly. Resume your original position.
Caution
Relax during the motions and make sure you feel no pain.

## Slide 3
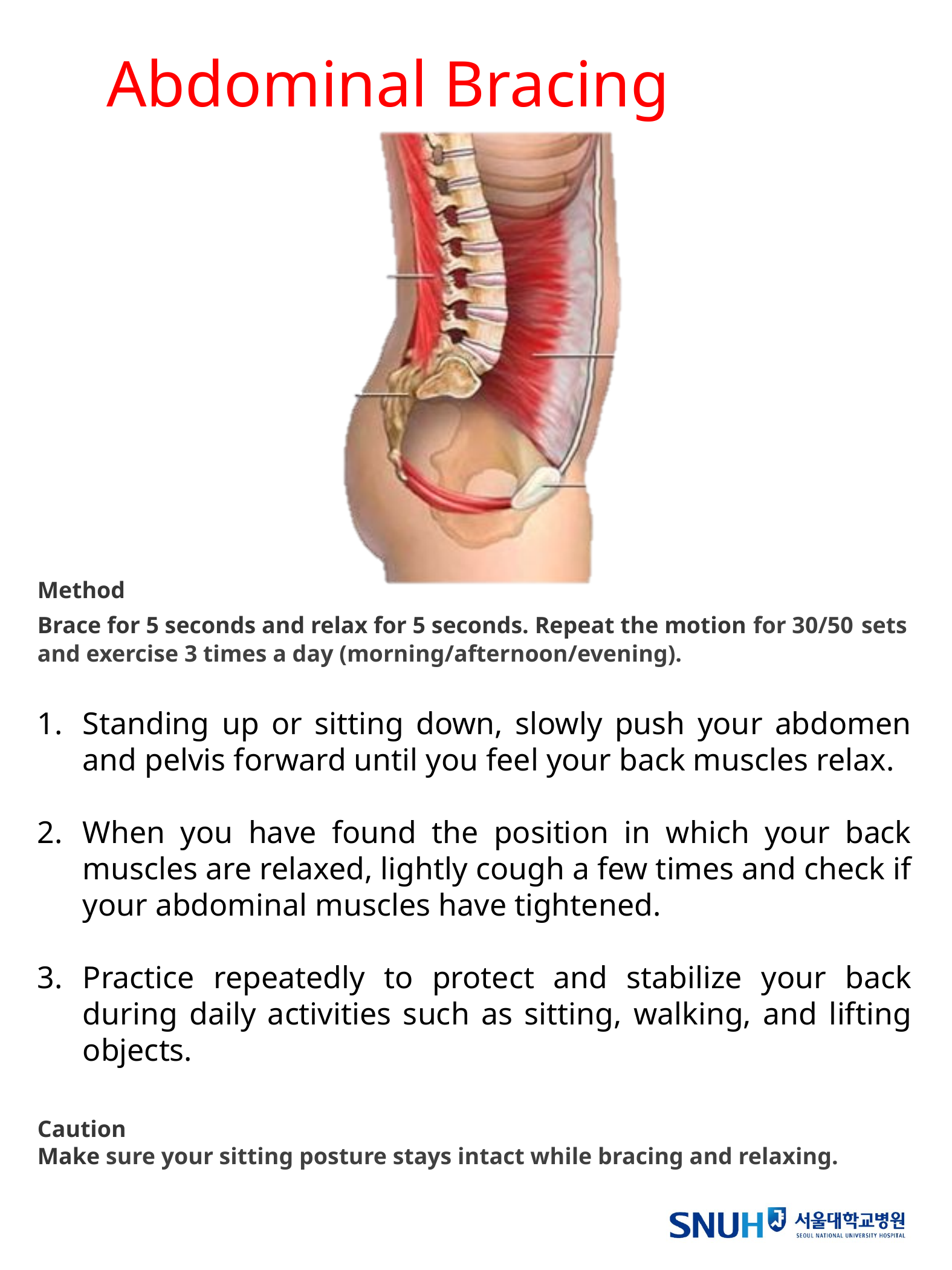

Abdominal Bracing
Method
Brace for 5 seconds and relax for 5 seconds. Repeat the motion for 30/50 sets and exercise 3 times a day (morning/afternoon/evening).
Standing up or sitting down, slowly push your abdomen and pelvis forward until you feel your back muscles relax.
When you have found the position in which your back muscles are relaxed, lightly cough a few times and check if your abdominal muscles have tightened.
Practice repeatedly to protect and stabilize your back during daily activities such as sitting, walking, and lifting objects.
Caution
Make sure your sitting posture stays intact while bracing and relaxing.

## Slide 4
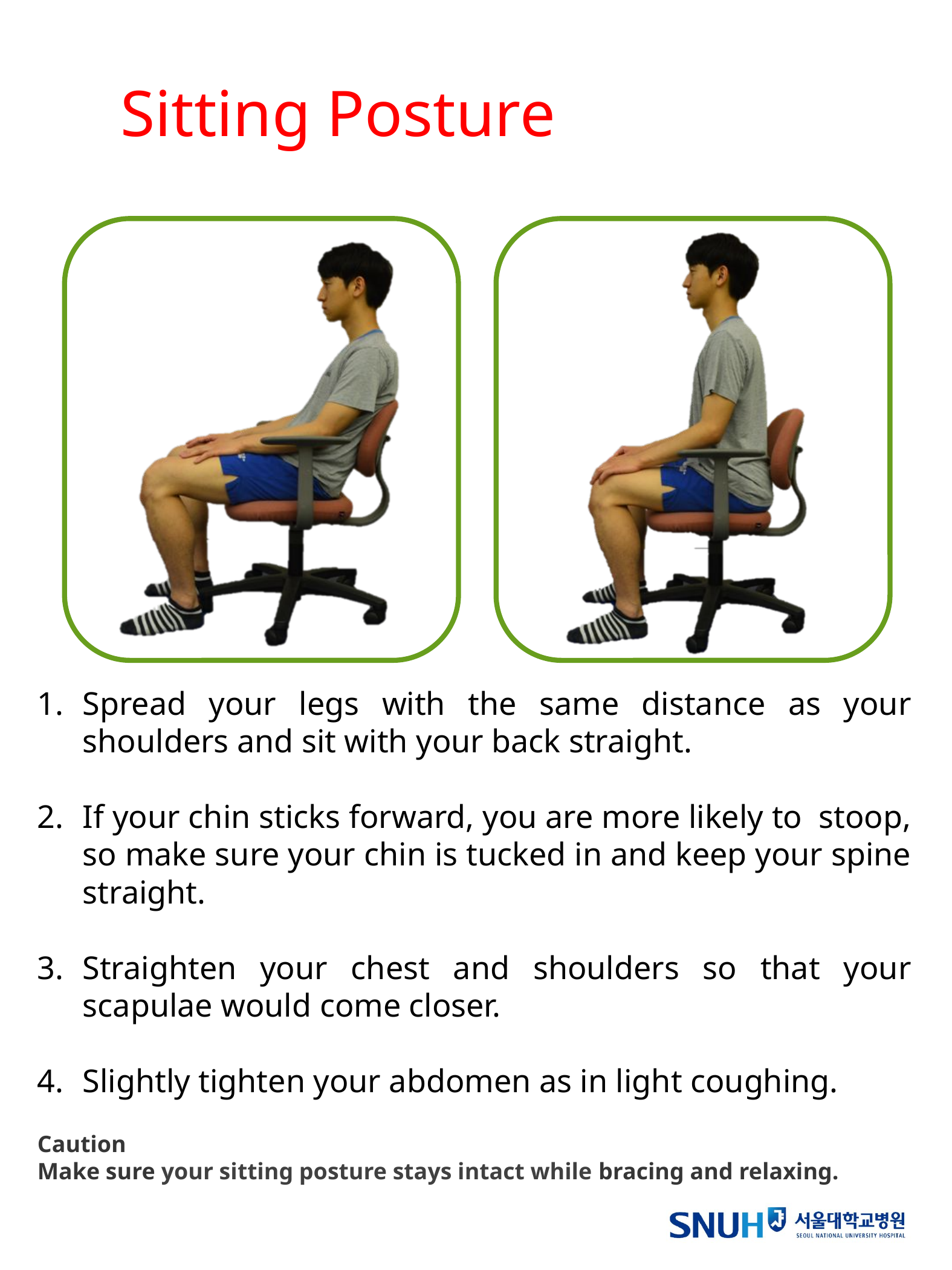

Sitting Posture
Spread your legs with the same distance as your shoulders and sit with your back straight.
If your chin sticks forward, you are more likely to stoop, so make sure your chin is tucked in and keep your spine straight.
Straighten your chest and shoulders so that your scapulae would come closer.
Slightly tighten your abdomen as in light coughing.
Caution
Make sure your sitting posture stays intact while bracing and relaxing.

## Slide 5
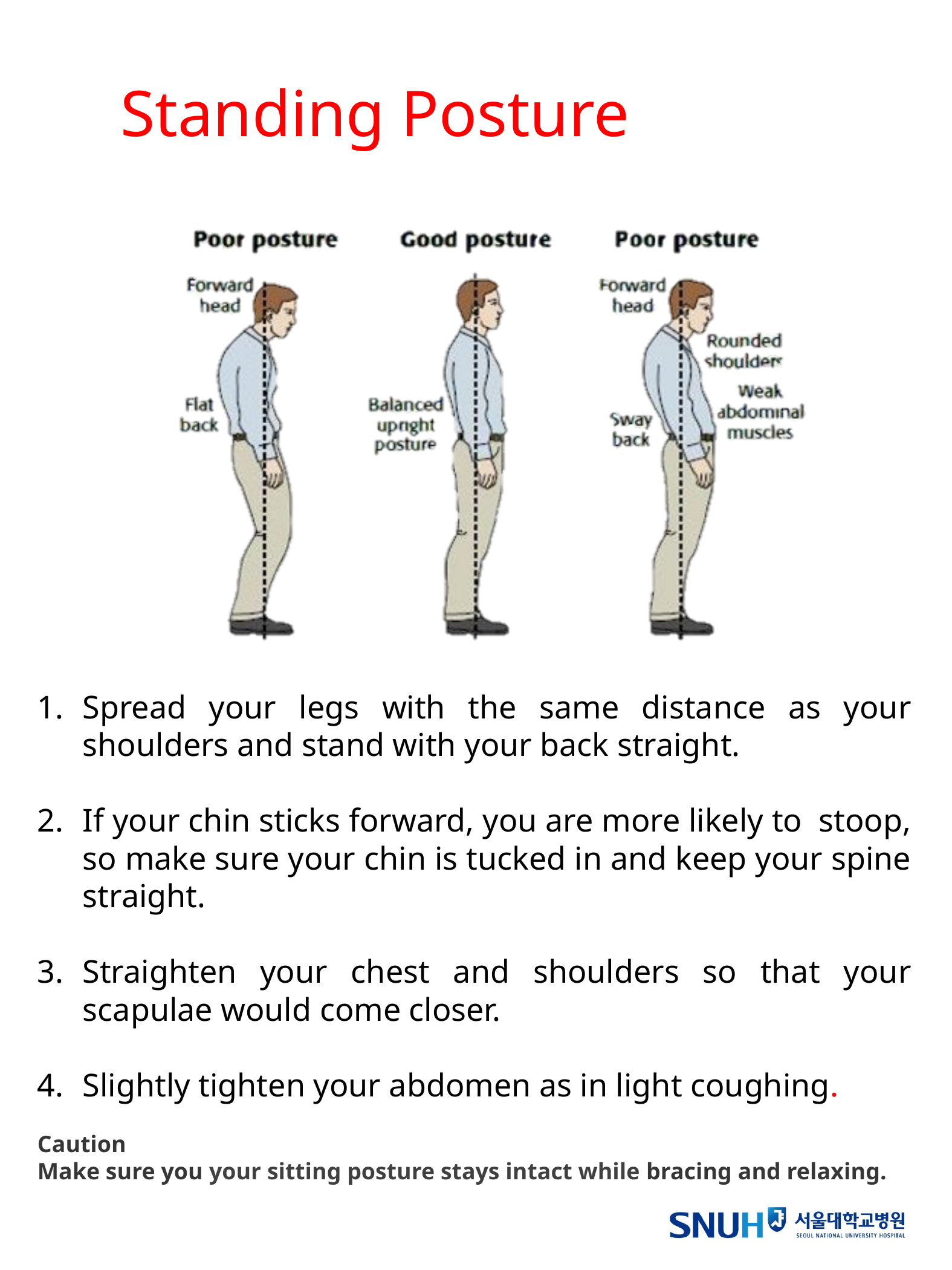

Standing Posture
Spread your legs with the same distance as your shoulders and stand with your back straight.
If your chin sticks forward, you are more likely to stoop, so make sure your chin is tucked in and keep your spine straight.
Straighten your chest and shoulders so that your scapulae would come closer.
Slightly tighten your abdomen as in light coughing.
Caution
Make sure you your sitting posture stays intact while bracing and relaxing.

## Slide 6
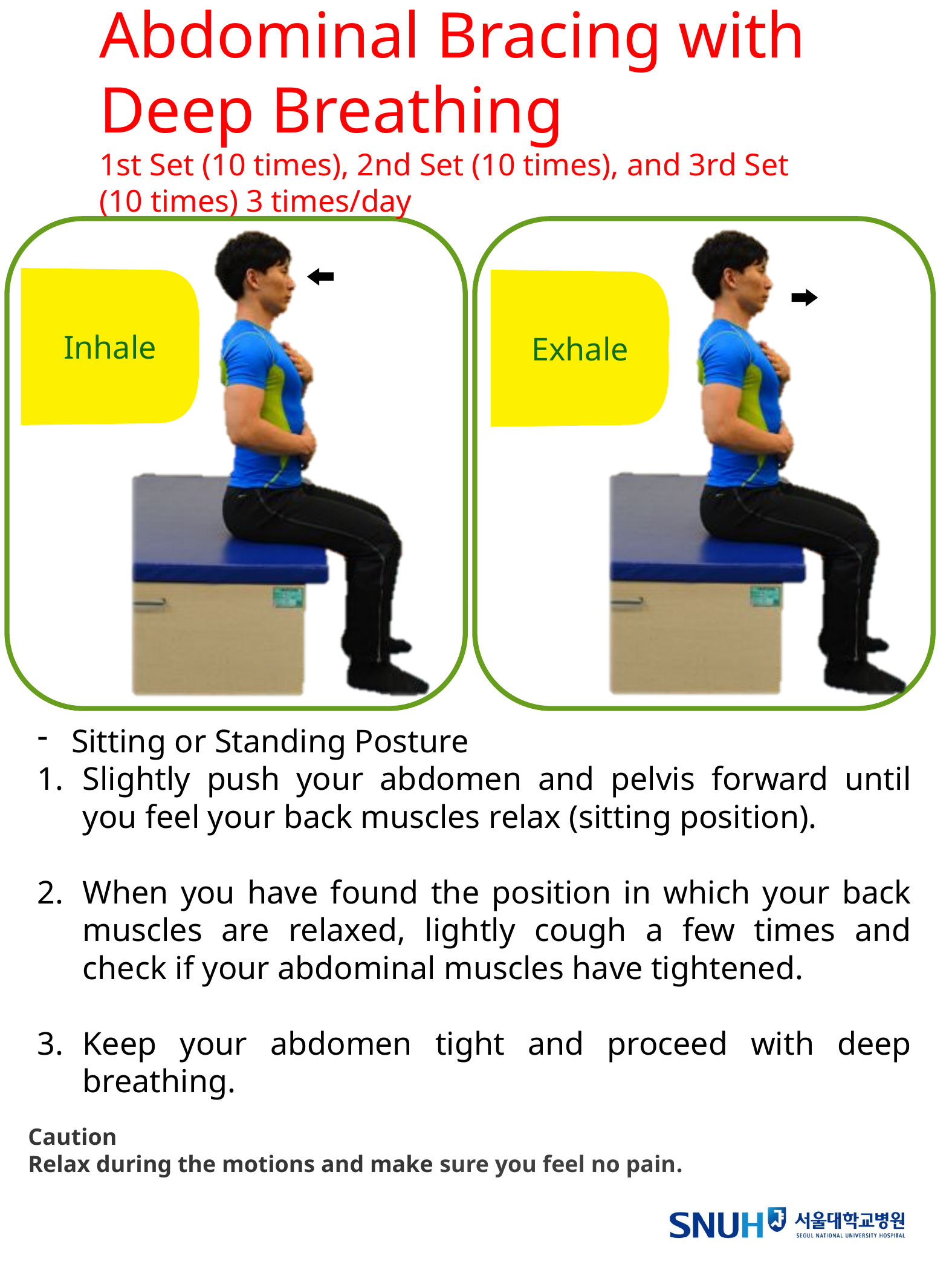

Abdominal Bracing with Deep Breathing
1st Set (10 times), 2nd Set (10 times), and 3rd Set (10 times) 3 times/day
Inhale
Caution
Relax during the motions and make sure you feel no pain.
Exhale
Sitting or Standing Posture
Slightly push your abdomen and pelvis forward until you feel your back muscles relax (sitting position).
When you have found the position in which your back muscles are relaxed, lightly cough a few times and check if your abdominal muscles have tightened.
Keep your abdomen tight and proceed with deep breathing.

## Slide 7
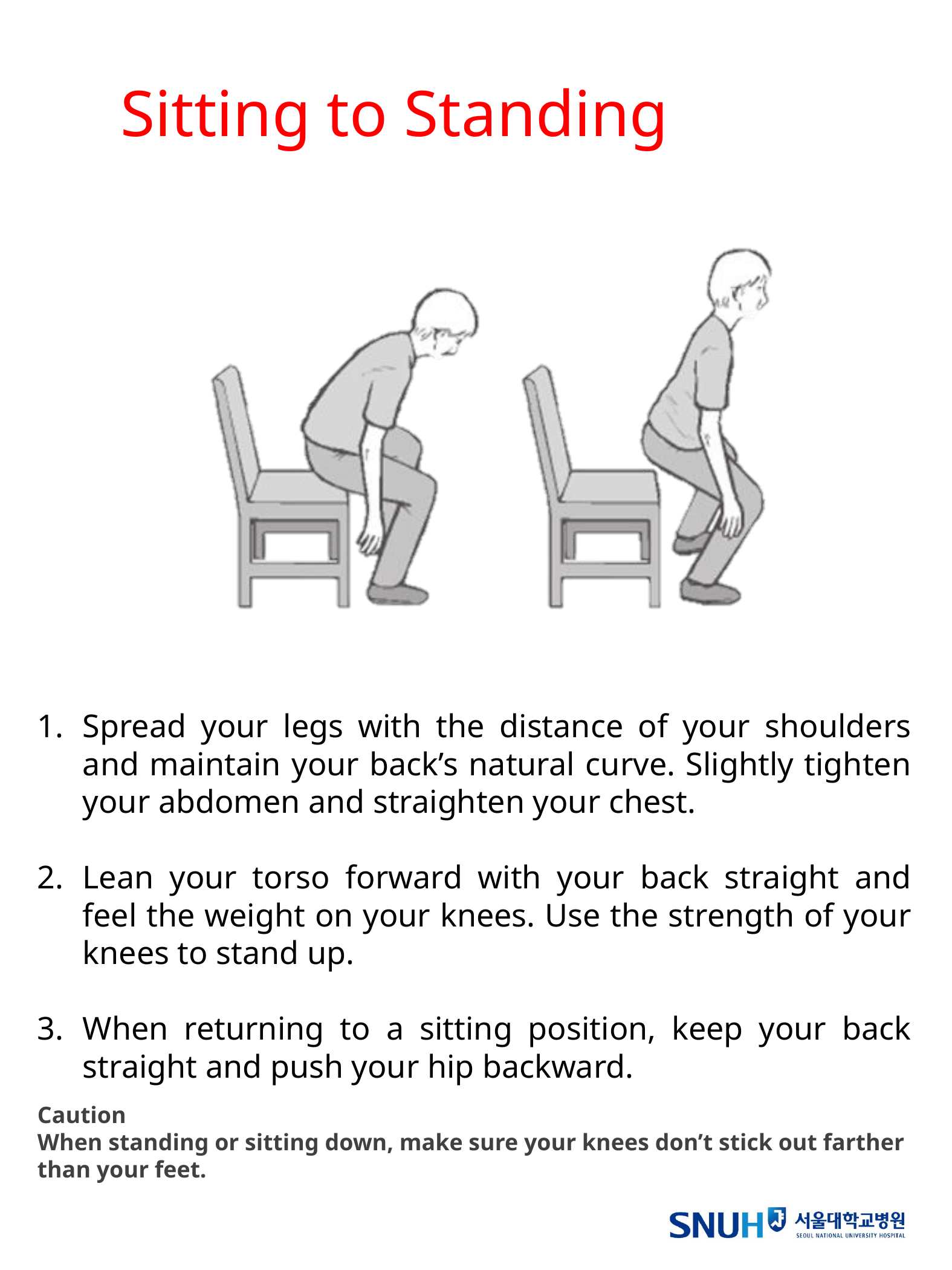

Sitting to Standing
Spread your legs with the distance of your shoulders and maintain your back’s natural curve. Slightly tighten your abdomen and straighten your chest.
Lean your torso forward with your back straight and feel the weight on your knees. Use the strength of your knees to stand up.
When returning to a sitting position, keep your back straight and push your hip backward.
Caution
When standing or sitting down, make sure your knees don’t stick out farther than your feet.

## Slide 8
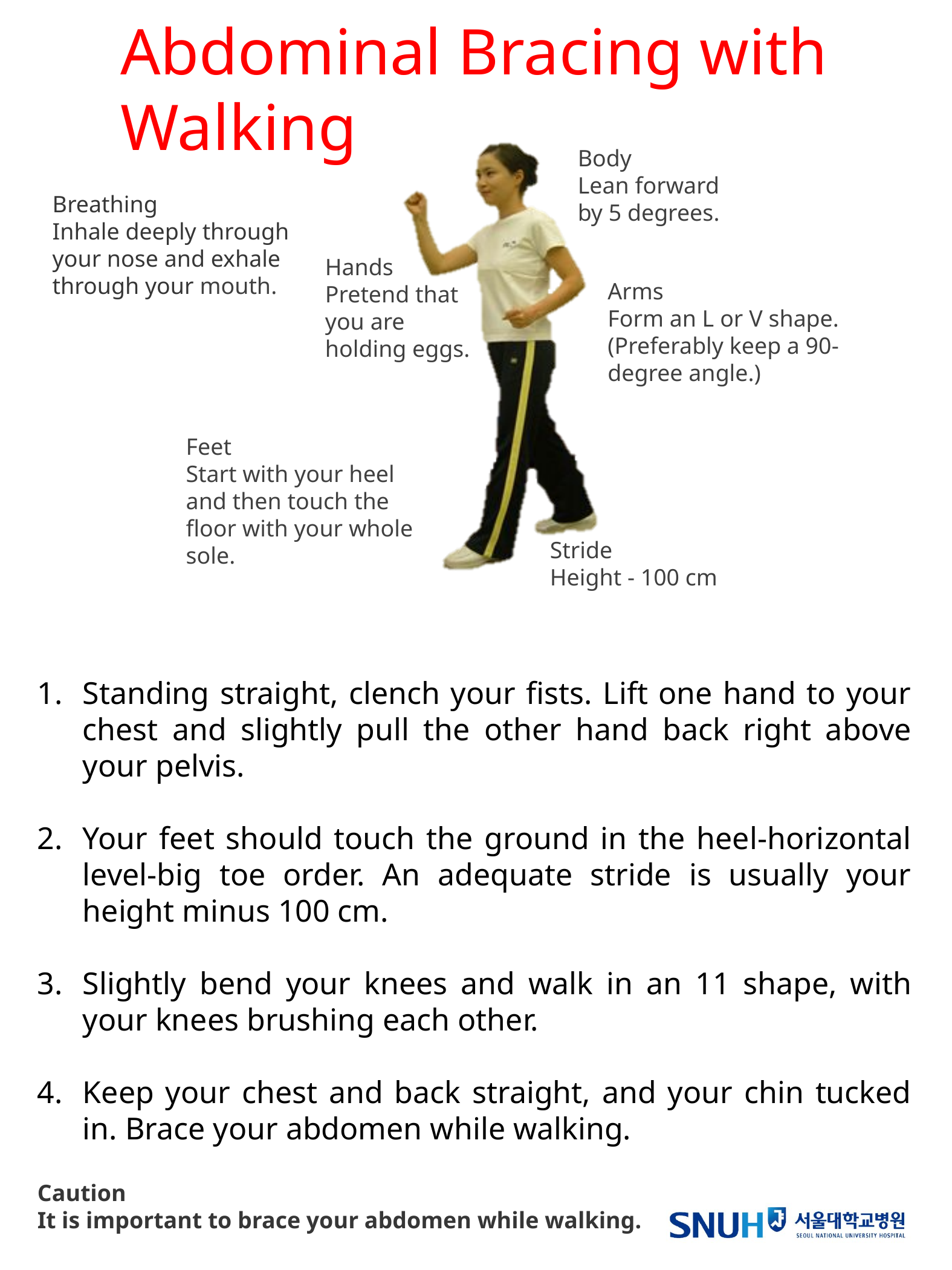

Abdominal Bracing with Walking
Body
Lean forward by 5 degrees.
Breathing
Inhale deeply through your nose and exhale through your mouth.
Hands
Pretend that you are holding eggs.
Arms
Form an L or V shape.
(Preferably keep a 90-degree angle.)
Feet
Start with your heel and then touch the floor with your whole sole.
Stride
Height - 100 cm
Standing straight, clench your fists. Lift one hand to your chest and slightly pull the other hand back right above your pelvis.
Your feet should touch the ground in the heel-horizontal level-big toe order. An adequate stride is usually your height minus 100 cm.
Slightly bend your knees and walk in an 11 shape, with your knees brushing each other.
Keep your chest and back straight, and your chin tucked in. Brace your abdomen while walking.
Caution
It is important to brace your abdomen while walking.

## Slide 9
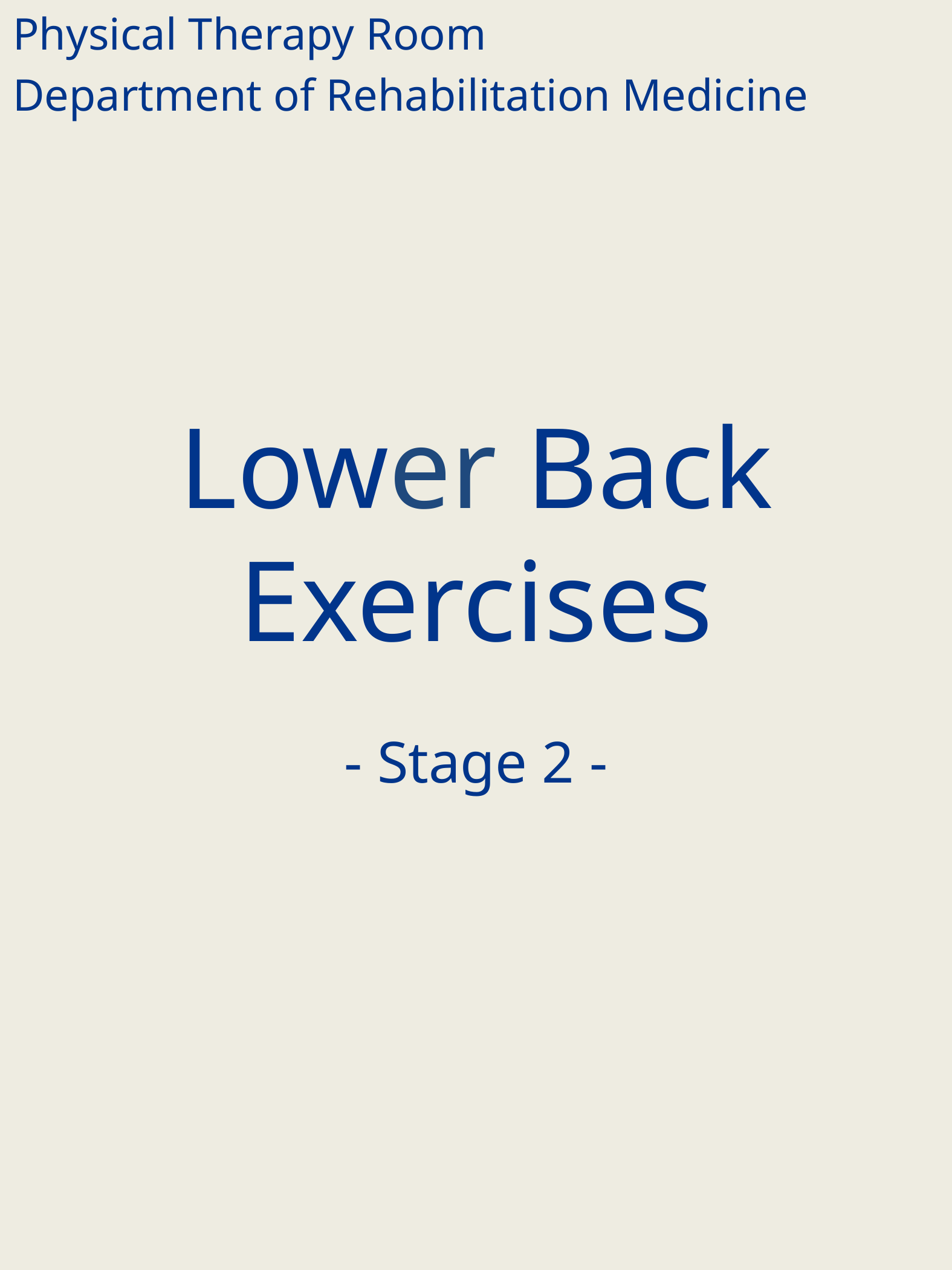

Physical Therapy Room
Department of Rehabilitation Medicine
# Lower Back Exercises
- Stage 2 -

## Slide 10
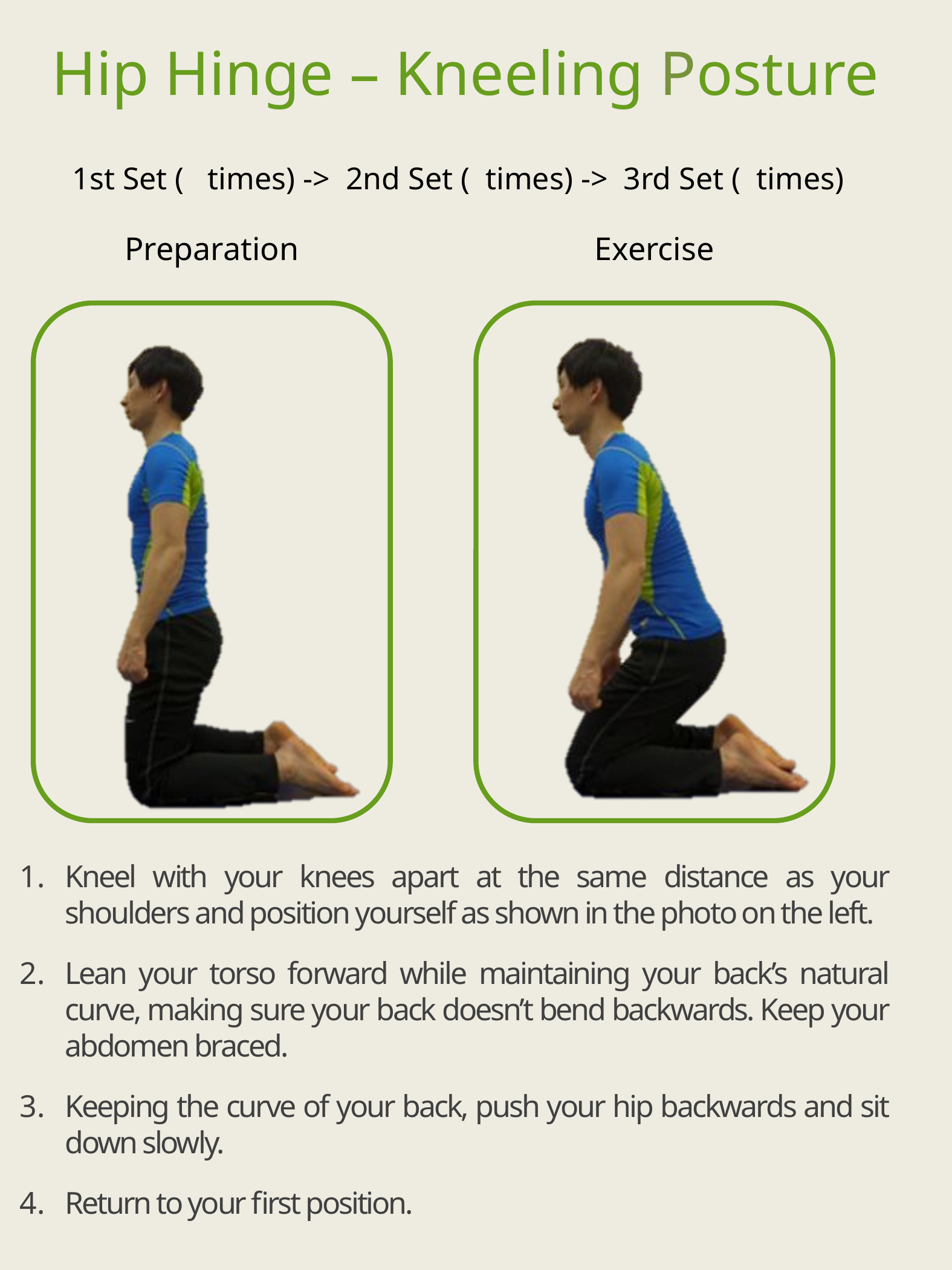

Hip Hinge – Kneeling Posture
1st Set ( times) -> 2nd Set ( times) -> 3rd Set ( times)
Preparation
Exercise
Kneel with your knees apart at the same distance as your shoulders and position yourself as shown in the photo on the left.
Lean your torso forward while maintaining your back’s natural curve, making sure your back doesn’t bend backwards. Keep your abdomen braced.
Keeping the curve of your back, push your hip backwards and sit down slowly.
Return to your first position.

## Slide 11
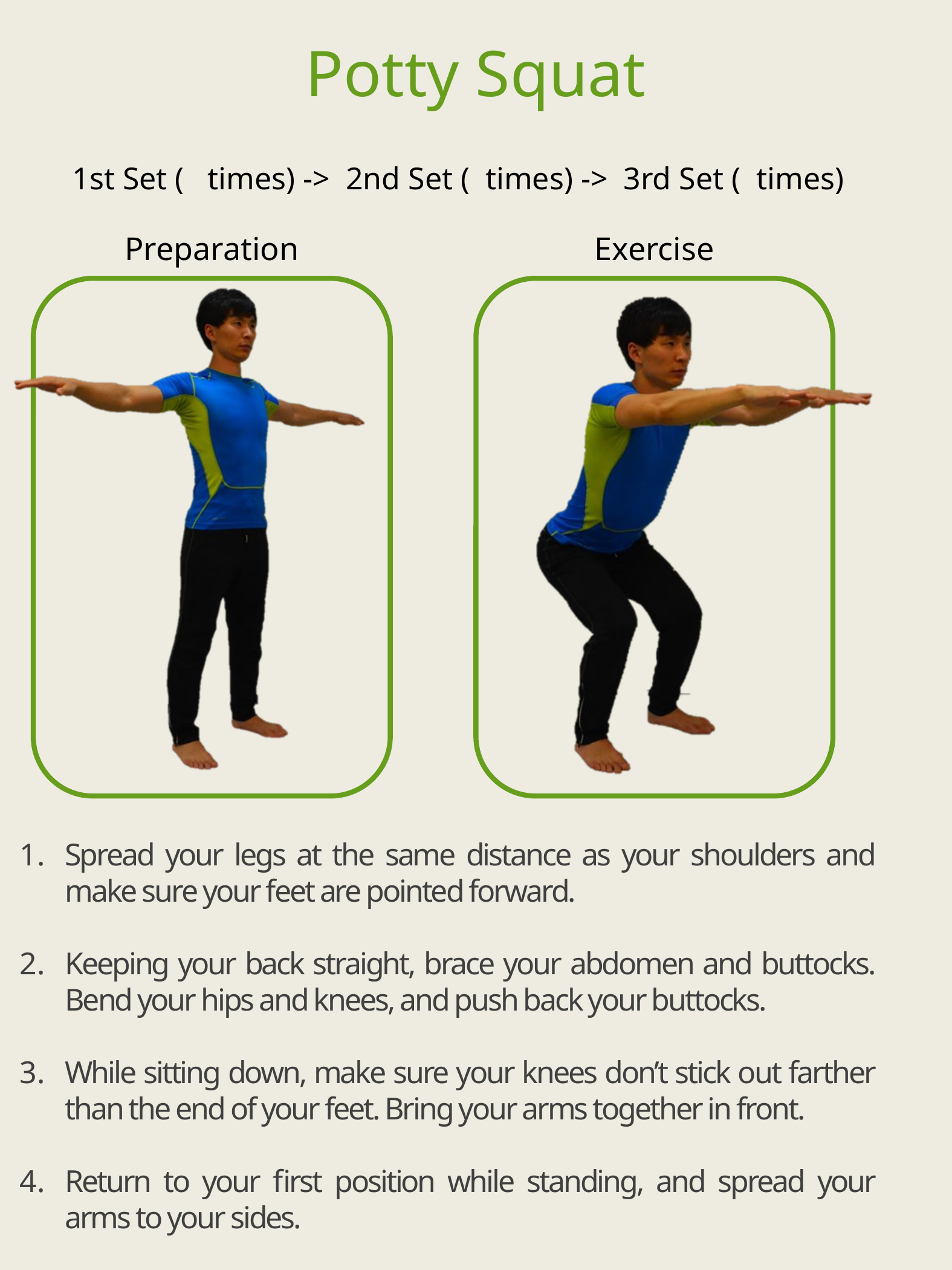

Potty Squat
1st Set ( times) -> 2nd Set ( times) -> 3rd Set ( times)
Preparation
Exercise
Spread your legs at the same distance as your shoulders and make sure your feet are pointed forward.
Keeping your back straight, brace your abdomen and buttocks. Bend your hips and knees, and push back your buttocks.
While sitting down, make sure your knees don’t stick out farther than the end of your feet. Bring your arms together in front.
Return to your first position while standing, and spread your arms to your sides.

## Slide 12
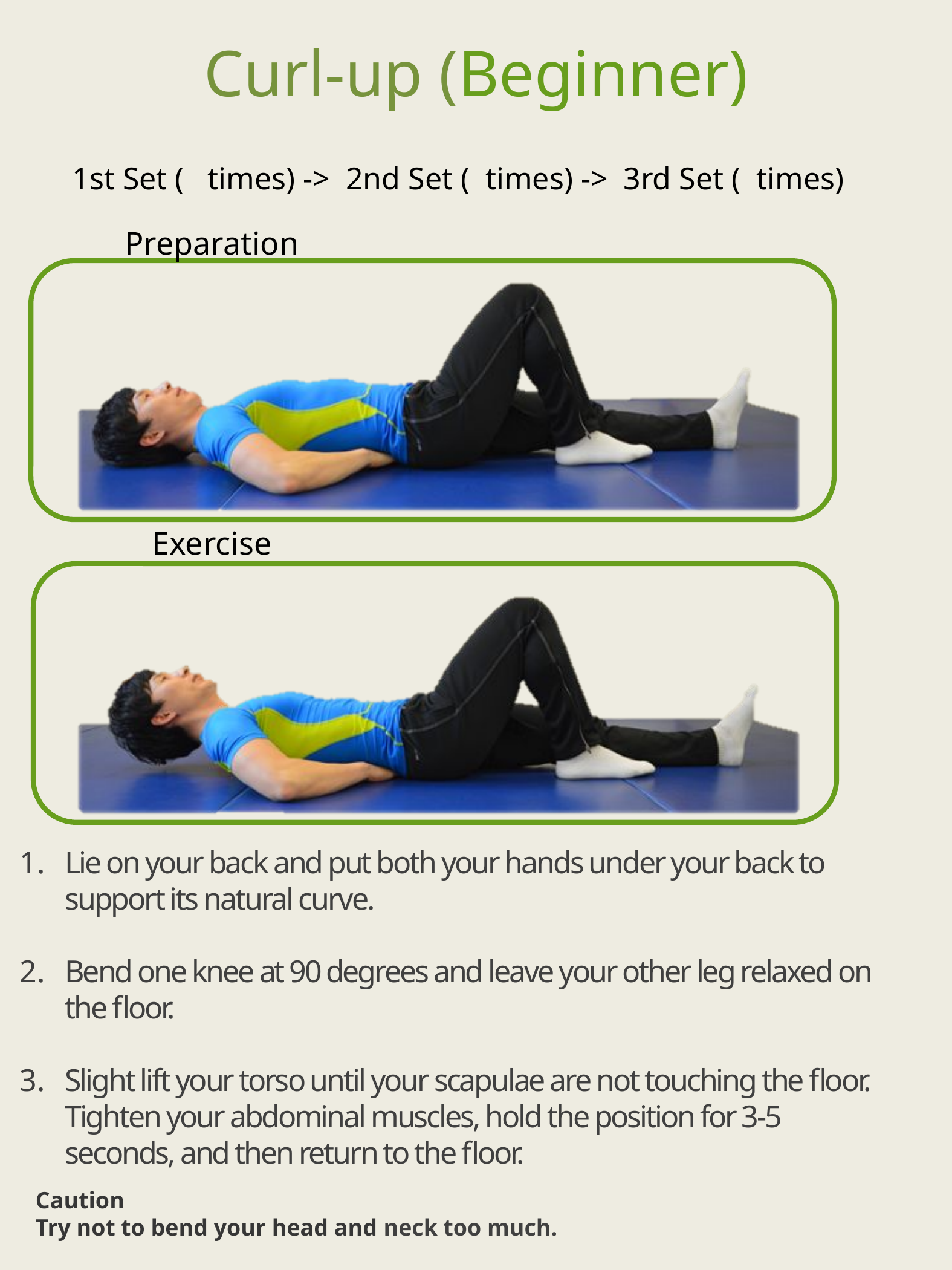

Curl-up (Beginner)
1st Set ( times) -> 2nd Set ( times) -> 3rd Set ( times)
Preparation
Exercise
Lie on your back and put both your hands under your back to support its natural curve.
Bend one knee at 90 degrees and leave your other leg relaxed on the floor.
Slight lift your torso until your scapulae are not touching the floor. Tighten your abdominal muscles, hold the position for 3-5 seconds, and then return to the floor.
Caution
Try not to bend your head and neck too much.

## Slide 13
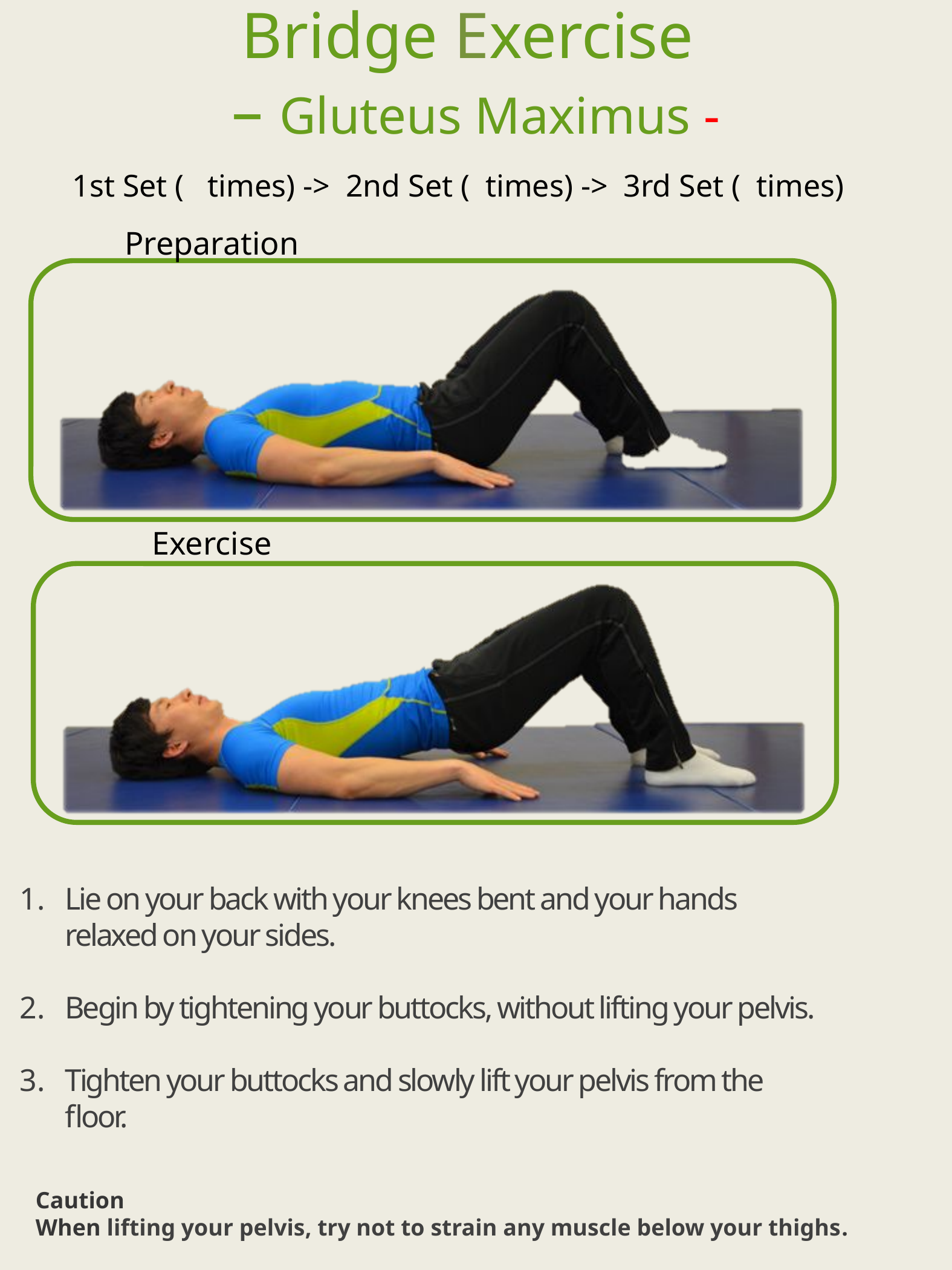

Bridge Exercise
– Gluteus Maximus -
1st Set ( times) -> 2nd Set ( times) -> 3rd Set ( times)
Preparation
Exercise
Lie on your back with your knees bent and your hands relaxed on your sides.
Begin by tightening your buttocks, without lifting your pelvis.
Tighten your buttocks and slowly lift your pelvis from the floor.
Caution
When lifting your pelvis, try not to strain any muscle below your thighs.

## Slide 14
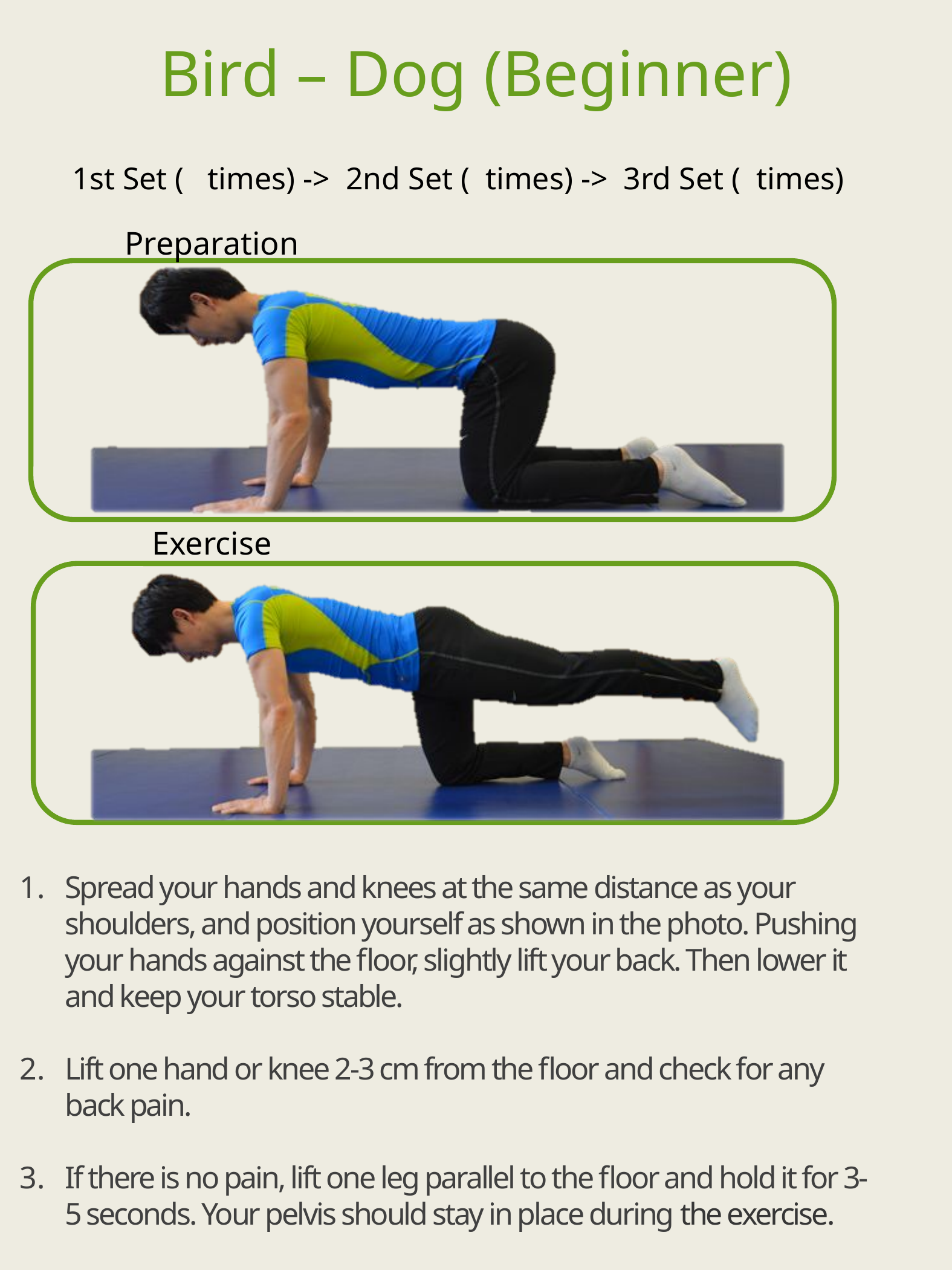

Bird – Dog (Beginner)
1st Set ( times) -> 2nd Set ( times) -> 3rd Set ( times)
Preparation
Exercise
Spread your hands and knees at the same distance as your shoulders, and position yourself as shown in the photo. Pushing your hands against the floor, slightly lift your back. Then lower it and keep your torso stable.
Lift one hand or knee 2-3 cm from the floor and check for any back pain.
If there is no pain, lift one leg parallel to the floor and hold it for 3-5 seconds. Your pelvis should stay in place during the exercise.

## Slide 15
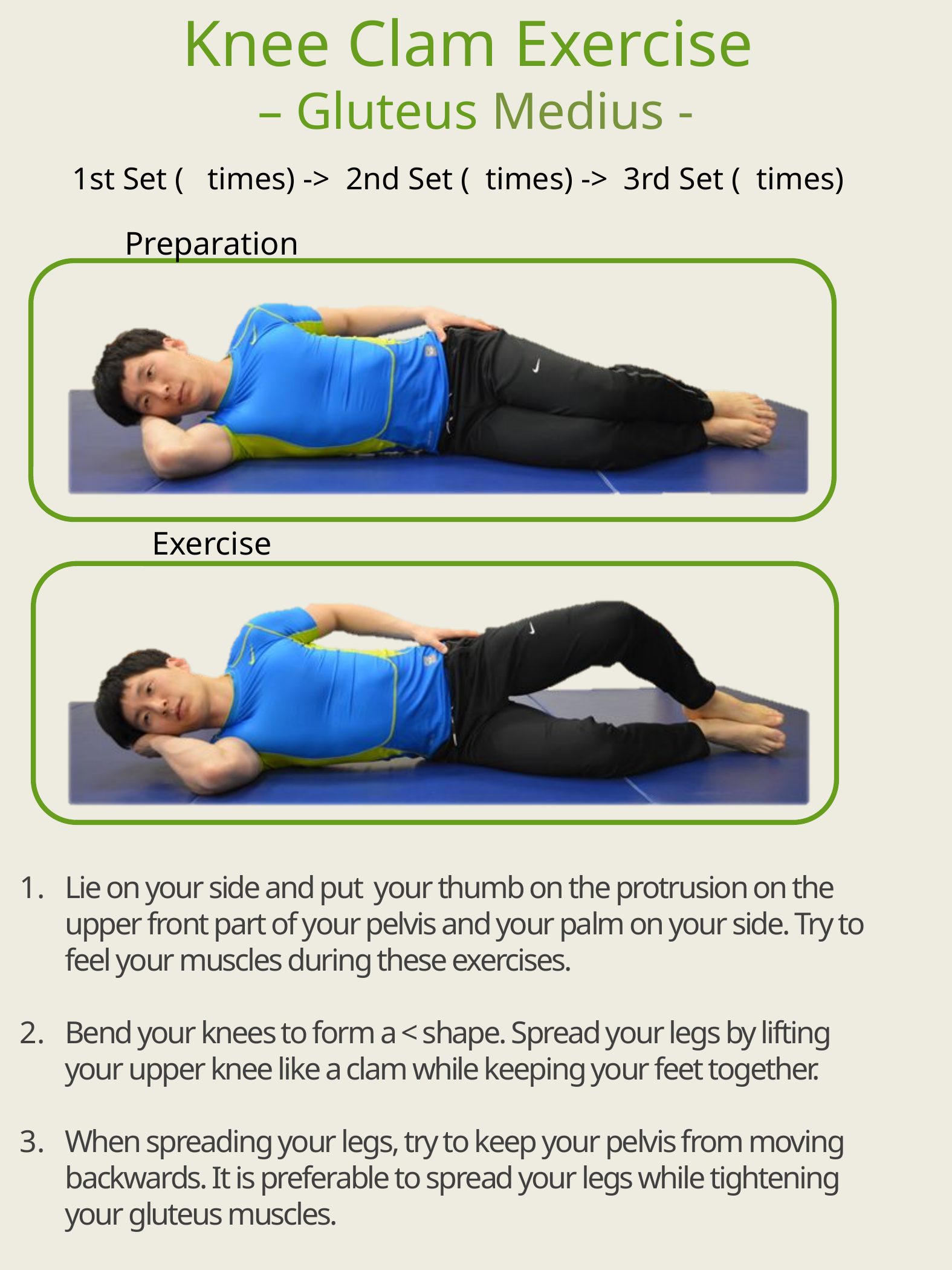

Knee Clam Exercise
– Gluteus Medius -
1st Set ( times) -> 2nd Set ( times) -> 3rd Set ( times)
Preparation
Exercise
Lie on your side and put your thumb on the protrusion on the upper front part of your pelvis and your palm on your side. Try to feel your muscles during these exercises.
Bend your knees to form a < shape. Spread your legs by lifting your upper knee like a clam while keeping your feet together.
When spreading your legs, try to keep your pelvis from moving backwards. It is preferable to spread your legs while tightening your gluteus muscles.

## Slide 16
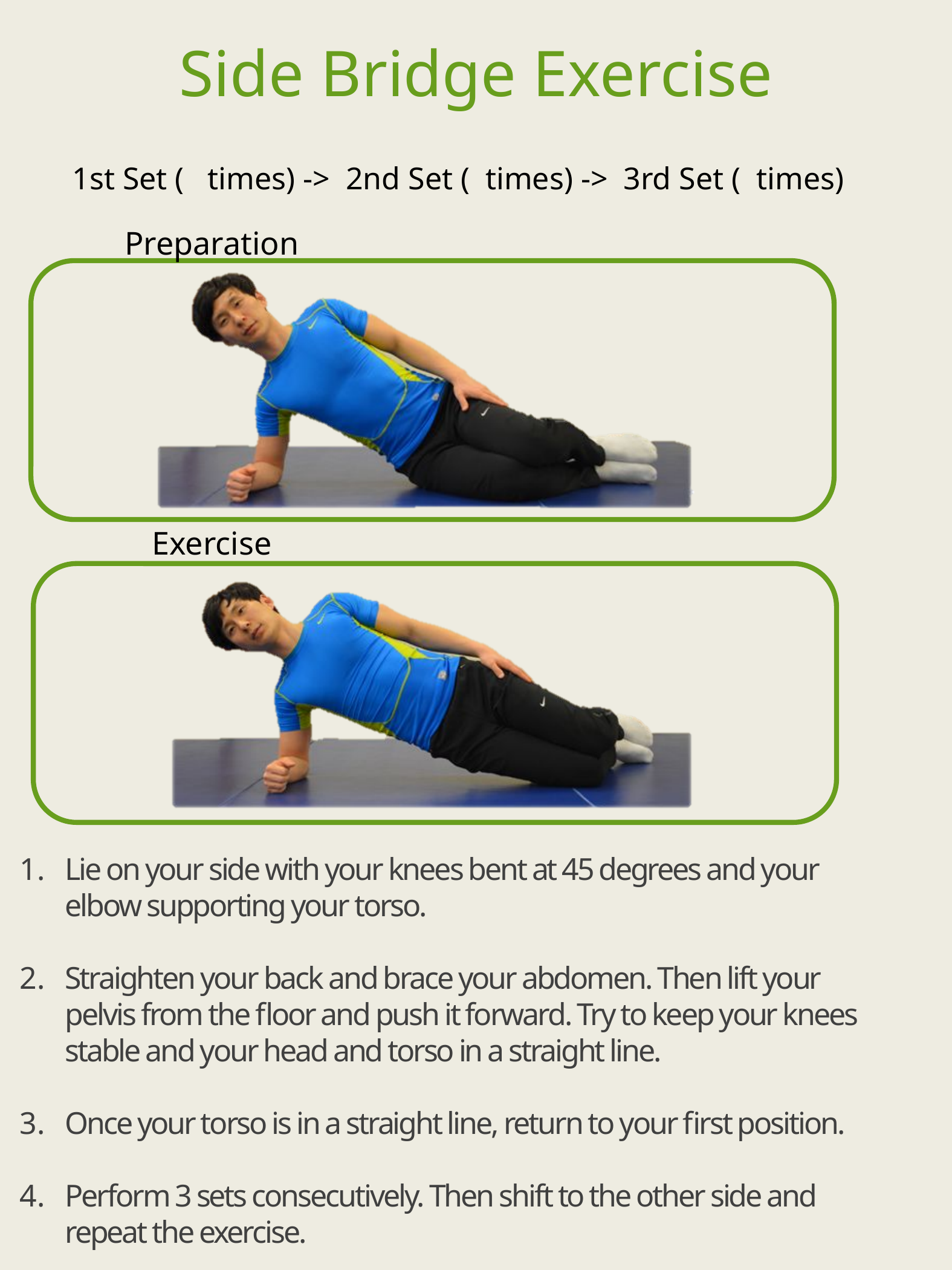

Side Bridge Exercise
1st Set ( times) -> 2nd Set ( times) -> 3rd Set ( times)
Preparation
Exercise
Lie on your side with your knees bent at 45 degrees and your elbow supporting your torso.
Straighten your back and brace your abdomen. Then lift your pelvis from the floor and push it forward. Try to keep your knees stable and your head and torso in a straight line.
Once your torso is in a straight line, return to your first position.
Perform 3 sets consecutively. Then shift to the other side and repeat the exercise.
